# Smaller is stronger: topological load-bearing of crumpled 2D macromolecule

**DOI:** 10.1093/nsr/nwag305

**Published:** 2026-05-27

**Authors:** Runze Liang, Kai Kang, Huichao Liu, Yingbo Yan, Yan Chen, Yilun Liu

**Affiliations:** Laboratory for Multiscale Mechanics and Medical Science, SV LAB, School of Aerospace, Xi’an Jiaotong University, Xi’an 710049, China; Laboratory for Multiscale Mechanics and Medical Science, SV LAB, School of Aerospace, Xi’an Jiaotong University, Xi’an 710049, China; Laboratory for Multiscale Mechanics and Medical Science, SV LAB, School of Aerospace, Xi’an Jiaotong University, Xi’an 710049, China; Laboratory for Multiscale Mechanics and Medical Science, SV LAB, School of Aerospace, Xi’an Jiaotong University, Xi’an 710049, China; Laboratory for Multiscale Mechanics and Medical Science, SV LAB, School of Aerospace, Xi’an Jiaotong University, Xi’an 710049, China; Laboratory for Multiscale Mechanics and Medical Science, SV LAB, School of Aerospace, Xi’an Jiaotong University, Xi’an 710049, China

**Keywords:** 2D macromolecules, topological load-bearing, size effect, confined mechanics, structure–property relation

## Abstract

Two-dimensional (2D) macromolecules are atomically thin materials capable of forming crumpled configurations with complex topologies, defining a new paradigm in macromolecular mechanics. Here, we unveil a universal negative size effect, where smaller sheets yield substantially stronger load-bearing capabilities than larger ones. Coarse-grained molecular dynamics simulations demonstrate a negative scaling between compression pressure or modulus and the Föppl–von Kármán number, with the power index determined by crumpling density but independent of material type. Energy analysis indicates that smaller sheets form dense ridge networks with minimal self-folding, enabling efficient load transfer and energy absorption. During densification, a constant ridge-to-vertex increment ratio of 1.5 preserves the superior ridge density of small sheets. Experiments on paper, aluminum foil, polydimethylsiloxane (PDMS), and silicone rubber confirm this behavior across disparate length scales and across material classes. This work reveals the mechanics underlying size-dependent crumpling in 2D macromolecules and provides principles for designing structural metamaterials with tunable load-bearing characteristics.

## Teaser

Crumpled thin sheets show a counterintuitive size effect—smaller balls carry greater loads at equal density by forming tighter fold networks, opening routes to lightweight high-performance materials.

## INTRODUCTION

Two-dimensional (2D) macromolecules are a transformative material class revolutionizing understanding of structure–property relationships across molecular-to-macroscopic scales. These atomically thin, covalently bonded networks encompass diverse materials (from graphene and transition metal dichalcogenides to biological membranes and synthetic polymer sheets) with exceptional in-plane properties and inherent flexibility for complex three-dimensional (3D) configurational transformations [1,2]. Unlike conventional 3D materials, 2D macromolecules spontaneously transition from flat to crumpled architectures under environmental stimuli, 3D confinement, or processing conditions [3,4]. This universal crumpling propensity transcends simple geometric transformation, redefining mechanical behavior, electronic transport, surface interactions, and biological function across nearly all application domains. Ubiquitous from solution-based molecular interactions [5,6] and industrial composite processing [7,8] to biological processes (e.g. insect wing morphogenesis [9], erythrocyte deformation [10,11], and neural tissue architecture [12,13]), this phenomenon endows crumpled 2D macromolecules with emergent properties that transcend molecular components. These materials thus constitute a distinct class with unique structure–property relationships, requiring novel theoretical frameworks and design principles [14].

The resulting crumpled particles exhibit emergent ‘topological load-bearing’ characteristics distinct from conventional continuum mechanics responses, laying the foundation for 2D macromolecule mechanics—an emerging interdisciplinary field bridging molecular science, statistical physics, and continuum mechanics [15,16]. This field reflects a paradigm shift in materials behavior: unlike traditional materials, whose mechanical properties stem primarily from chemical composition and crystalline structure, crumpled 2D macromolecules derive performance from topological configurations emerging via stochastic folding and bending events [5,17]. Crumpling transforms flat 2D structures into complex 3D architectures, where macroscopic mechanical behavior depends on the spatial arrangement and dynamic evolution of microscopic topological features rather than predictable continuum laws [18].

Comprehensive investigations have identified four primary topological microstructures during conformational densification: localized bending, sharp folding [19,20], extended ridges [21,22], and cones [23,24], which collectively orchestrate the load-bearing capacity and mechanical response of crumpled particles [25,26]. These microstructural elements critically determine the load-bearing capacities of crumpled particles [21], which typically follow an exponential scaling law analogous to porous foams [27–29]: load-bearing force ($F$) scales with the inverse of indenter distance (*H*) raised to an exponent $\alpha$, where $\alpha$ encapsulates the combined effects of compression mode and material properties [21,30,31]. Building on this relationship, a more refined empirical formulation incorporates additional material parameters—including the plane strain modulus $\bar{\,\,E} = {E}_0/( {1 - {\nu }^2} )$ (with Young’s modulus ${E}_0$ and Poisson ratio $\nu$) [21,31,32], as well as the film thickness ${t}_0$ and the crumpled particle’s radius $R$—yielding the equation:


(1) \begin{eqnarray*}
F = {\bar{\,\,E}{t}}_0^2{\left( {\frac{{2R}}{H}} \right)}^\alpha .
\end{eqnarray*}

While these empirical models effectively predict load-bearing capacities and facilitate material characterization [21,32], they fail to elucidate the mechanistic relationship between load-bearing response and topological microstructure evolution during densification.

Prior studies focus on individual ridge mechanics [22,33,34], with ridge length identified as a key compressive resistance determinant [21]. Extrapolating collective load-bearing behavior remains challenging due to complex inter-ridge interactions [35,36], encompassing friction and adhesion. While adhesion-induced stacking enhances compressive modulus [37], this effect is inherently tied to the internal ridge network. Advanced characterization (such as X-ray tomography and energy landscape analysis) characterizes network spatial distribution [37,38], but the mechanisms governing their evolution remain elusive [31,32]. Critical parameters such as sheet size and crumpled density directly modulate load-bearing characteristics, potentially leading to significant deviations from established scaling laws [20,39]. The underlying mechanisms governing size-dependent load-bearing capacity of crumpled particles during conformational densification remain inadequately explored and understood.

To systematically address these knowledge gaps, we used coarse-grained molecular dynamics (CGMD) simulations to study conformational densification of 2D macromolecules under 3D confinement—revealing an unexpected negative size effect: smaller sheets exhibit higher load-bearing capacity. Through energy landscape and microstructural analyses, we identify the dominant factors governing load-bearing capacity during densification. Additionally, we validate our findings through experimental investigations across diverse thin film materials.

## RESULTS

### Negative size effect

CGMD simulations were performed to elucidate the microstructural mechanisms governing the mechanics of crumpled 2D macromolecules under 3D confinement. Given that prior work has shown sheet size and crumpled density both significantly influence the internal structure of crumpled particles [40,41], these parameters are often conflated in literature [39]. Herein, we systematically isolated their individual contributions to the mechanical response of crumpled particles.

**Figure 1.**
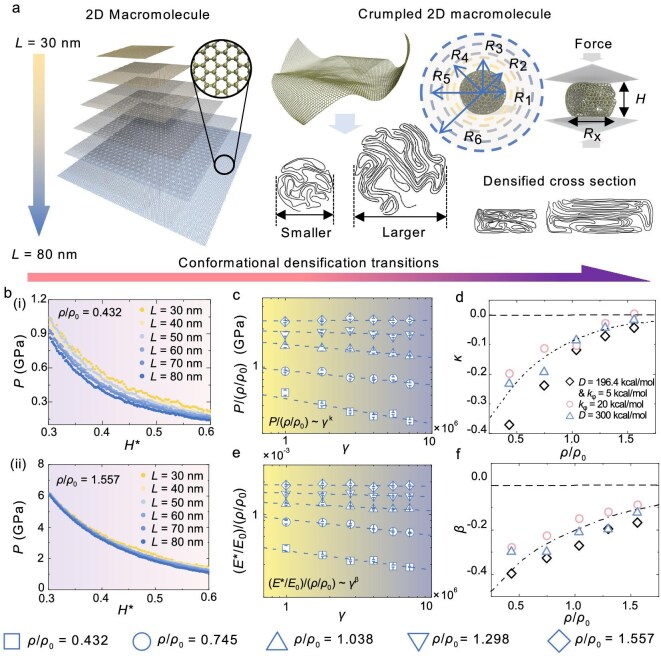
Negative size effect in 2D macromolecules revealed through CGMD simulations. (a) Coarse-grained (CG) models of discrete honeycomb lattices structures (*L* = 30–80 nm). (b) Loading *P–*${H}^*$ curves at varying initial relative densities (i: $\rho /{\rho }_0 = 0.432$, ii: $\rho /{\rho }_0 = 1.557$). (c) Scaling relationship between $P/(\rho /{\rho }_0)$ and $\gamma$ at ${H}^*$ = 0.4, following the power law $P/(\rho /{\rho }_0)\ \sim \ {\gamma }^\kappa$. (d) Variation of scaling exponent $\kappa$ as a function of $\rho /{\rho }_0$ across three distinct material configurations. (e) Relation between $( {{E}^*/{E}_0} )/( {\rho /{\rho }_0} )$and *γ* following a scaling relationship with power exponent β. (f) Variation of β as a function of $\rho /{\rho }_0$ across different material parameters. In (d and f), dotted lines indicate trend lines, while horizontal dashed lines represent the limiting scenario where crumpled particles approximate solid homogeneous materials. In (c and e), interaction parameters are *D*_0_ = 196.38 kcal/mol, ${k}_\theta$ = 409.4 kcal/mol, ${k}_\varphi$ = 5 kcal/mol, and *ε* = 0.001 kcal/mol. (Here, ${D}_0$ denotes the bond dissociation energy, ${k}_\theta$ the bond angle bending stiffness, ${k}_\varphi$ the dihedral torsional stiffness, and $\varepsilon$ the non-bonded interaction well depth; detailed functional forms and numerical values are provided in Table S1).

Our simulation framework used self-avoiding 2D macromolecules discretized into honeycomb lattices structures, mimicking real thin films [30] (detailed definitions in Materials and Methods). The protocol included two stages (Fig. 1a, Figs S1 and S2, Videos S1 and S2): initial crumpling in a virtual spherical shell, followed by controlled compression between rigid plates. To establish consistent initial conditions across different sheet sizes, we precisely adjusted the confining radii to preserve a constant relative density $\rho /{\rho }_0$, where ${\rho }_0$ represents the 2D macromolecule density, and $\rho = \frac{m}{{4/3\pi {r}^3}}$ is the mean crumpled density after the initial crumpling process (Fig. 1a), where $m$ is the mass of the original flat film and $r$ is measured radius of the crumpled ball. Since the film mass remains constant, the relative density is governed by the volume change after the initial crumpling process, expressed as $\rho /{\rho }_0 = {V}_0/V = \frac{{3{t}_0{L}^2}}{{4\pi {R}^3}}$, where $R$ denotes the radius of the crumpled two-dimensional macromolecule. The densification level is defined as H* = *H*/*H*_0_, with *H* and *H*_0_ denote the current and initial end-to-end distances between the compression plates, respectively.

For a consistent comparative analysis across different sizes, the sheet dimension $L$ is normalized using the Föppl-von Kármán (FvK) number, $\gamma \equiv {E}_0{L}^2/D$[22,42], where ${E}_0$ and $D$ are the elastic modulus and bending stiffness of 2D macromolecules, respectively. To eliminate the effect of crumpled ball size on the load during load bearing and to characterize the load-bearing capacity, we define the nominal pressure, $P = F/( {\pi {R}^2} )$. The corresponding *P*–*H* relationship can be expressed,


(2) \begin{eqnarray*}
P = {E}^*{\left( {\frac{{2R}}{H}} \right)}^\alpha ,
\end{eqnarray*}

where ${E}^* = \bar{\,\,E}{t}_0^2/( {\pi {R}^2} )$ is denoted the load-bearing modulus, to facilitate comparison of the effective elastic modulus across different sheet sizes. As shown in Fig. 1b (average of 6 simulations per size), $P{\mathrm{\ }}$undergoes a rapid power-law increase with decreasing ${H}^{\mathrm{*}}$, indicating progressively enhanced compressive resistance at higher densification states. Remarkably, our systematic investigation across different sheet sizes reveals a consistent negative size effect—manifested as a systematic downward shift in *P*–${H}^*$ curves with increasing sheet size—independent of initial density $\rho /{\rho }_0$. This counterintuitive behavior becomes less pronounced at higher densification states, as evidenced by the convergence of load-bearing curves at higher $\rho /{\rho }_0$(Fig. 1b (ii)), suggesting diminished size sensitivity under very high densification.

To explore crumpled density’s impact on the negative size effect, we performed CGMD simulations across multiple relative densities. The *P*–$\gamma$ relationship under different $\rho /{\rho }_0$ follows an approximate scaling law (Fig. 1c):


(3) \begin{eqnarray*}
P/\left( {\rho /{\rho }_0} \right)\sim {\gamma }^\kappa ,
\end{eqnarray*}

where $\kappa$ characterizes load-bearing capacity’s sensitivity to the size effect. Our study consistently reveals negative $\kappa$ values, confirming the negative size effect. As $\rho /{\rho }_0$ increases, the value of $\kappa$ asymptotically approaches zero, indicating an enhanced packing ratio leading to solid-sphere-like behavior, which is independent of the size effect. Under these conditions, conformational transitions become increasingly constrained, leading to diminished size sensitivity. Notably, this density-dependent behavior of $\kappa$ remains consistent across various material parameters in our CG models, systematically converging towards zero with increasing $\rho /{\rho }_0$ (Fig. 1d), demonstrating a general density-modulated size effect regardless of intrinsic material properties.

Figure 1e illustrates the ${E}^* \\!-\\! \gamma$ relationship (see Fig. S3 for detailed fitting analysis), which also follows an approximate scaling law:


(4) \begin{eqnarray*}
\left( {\frac{{{E}^*}}{{{E}_0}}} \right)\Big/\left( {\frac{\rho }{{{\rho }_0}}} \right)\sim {\gamma }^\beta ,
\end{eqnarray*}

where $\beta$ quantifies the sensitivity of the load-bearing modulus to the size effect. Analysis across different densities reveals that β increases with $\rho /{\rho }_0$, approaching the limiting cases of a solid sphere ($\beta$ = 0) (Fig. 1f). This scaling relationship further confirms the negative size effect during conformational densification, with the magnitude intrinsically coupled to the relative density of the crumpled particles. Importantly, this negative size effect manifests as a characteristic feature of crumpled particles possessing complex porous architectures, progressively diminishing upon reaching certain structural densification thresholds (Fig. 1f). Although $\kappa$ and $\beta$ exhibit similar trends with relative density, they represent different physical quantities: $\kappa$ characterizes the scaling of the load-bearing response at a given ${H}^{\mathrm{*}}$, whereas $\beta$ quantifies the scaling of the effective load-bearing modulus.

Notably, our findings are in complete contrast to those reported by O. Bouaziz *et al.* [43], who observed positive size effects. This discrepancy arises from their experimental conditions, where larger sheet dimensions resulted in higher crumpled densities within fixed volumes, consequently yielding increased pressure and load-bearing modulus. Essentially, their analysis did not distinguish the independent contributions of sheet size and crumpled density. Our study elaborately controls crumpled density, thereby enabling a discrete examination of these two effects and revealing the true nature of size-dependent mechanical behavior in crumpled 2D macromolecules.

### Folded patterns and energy distributions

Our CGMD simulations reveal a universal negative size effect governing the load-bearing capacity of crumpled 2D macromolecules, intrinsically linked to conformational densification transitions. However, the complexity and stochasticity of topological microstructures formed during densification pose notable challenges for studying size-dependent phenomena via non-interventional experiments. Herein, we tackle these challenges with detailed CGMD simulations to uncover the fundamental mechanisms underlying this counterintuitive behavior.

**Figure 2.**
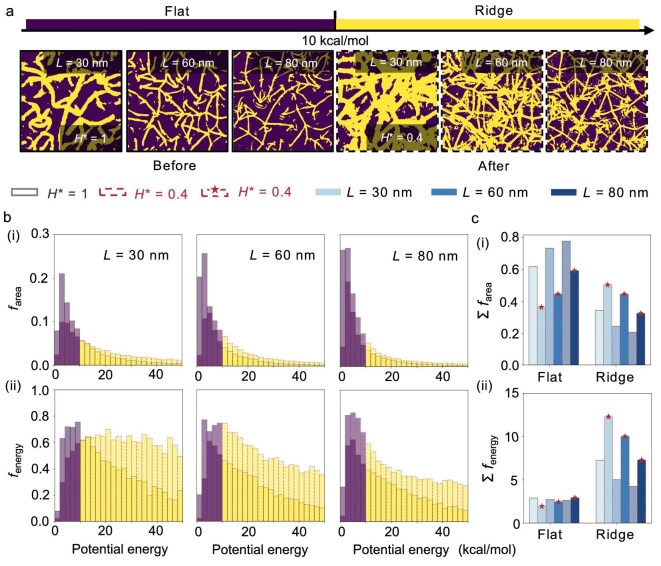
Energy landscapes and distribution patterns in crumpled 2D macromolecules. (a) Visualization of energy landscapes before compression (${H}^*$ = 1) and after compression (${H}^*$ = 0.4) for crumpled 2D macromolecules of different sizes (*L* = 30 nm, 60 nm and 80 nm). Ridge structures and planar regions are differentiated using a 10 kcal/mol energy threshold (The figures show local images). (b) Quantitative characterization through: (i) the energy probability distribution *f*_area_ and (ii) the local energy density *f*_energy_ before compression (${H}^*$ = 1) and after compression (${H}^*$ = 0.4). (c) Comparative analysis of ∑*f*_area_ and ∑*f*_energy_ in flat and ridge structures across different sheet sizes.

To clearly and concisely illustrate the underlying mechanism of load-bearing capacity, we consider $\rho /{\rho }_0 = 0.432$. Figure 2a presents energy landscapes mapped onto flattened films of different sizes (*L* = 30, 60, and 80 nm) before compression (${H}^{\mathrm{*}}$ = 1) and after compression (${H}^{\mathrm{*}}$ = 0.4) from CGMD simulations (${D}_0$ = 196.38 kcal/mol, ${k}_\theta$ = 409.4 kcal/mol, ${k}_\varphi$ = 5 kcal/mol, and $\varepsilon$ = 0.001 kcal/mol), where the topological microstructures are distinguished into ridge (yellow) and flat (purple) regions using a defined threshold energy (without loss of generality, a threshold value of 10 kcal/mol is adopted here, and the rationale for this choice is discussed in the Note S1). After compression, ridge areas expand systematically across various sheet sizes, forming increasingly complex networks characterized by bilateral expansion from primary ridges and dendritic branching patterns reminiscent of hierarchical tree structures. This systematic expansion of the ridge network with increasing load drives the crumpled structure toward more complex topological configurations. At the same time, the figure qualitatively shows that both before and after compression, smaller sheets exhibit a greater abundance of ridge structures.

To quantitatively analyze the distribution of ridge and flat regions within the crumpled particles, we examined the energy distributions (Fig. 2b) as well as their integrated values (Fig. 2c), where the solid outlines denote the state before compression and the dashed outlines denote the state after compression. Specifically, we analyzed two key parameters: ${f}_{{\mathrm{area}}}$, defined as the energy probability distribution, i.e. the fraction of beads or MD particles with a given potential energy; and ${f}_{{\mathrm{energy}}}$, defined as the local energy density, i.e. the product of the local potential energy and ${f}_{{\mathrm{area}}}$. The analysis of ${f}_{{\mathrm{area}}}$ first reveals a systematic evolution in the energy distribution: the ridge contribution exhibits a localized enhancement, as evidenced by the increase in histogram height in the high-energy region (Fig. 2b(i)). Furthermore, the ${f}_{{\mathrm{energy}}}$ of the ridge regions increases significantly after compression, accompanied by a pronounced decrease in the flat regions (Fig. 2b(ii) and c(ii)). This redistribution indicates rapid energy localization within the ridge structures, resulting in a substantial increase in local energy density at ridge sites. Therefore, our results establish ridges as the primary determinant of load-bearing capacity in crumpled particle systems, in agreement with previous studies showing that cumulative energy absorption in crumpled particles is predominantly concentrated within ridge networks, thereby reinforcing their critical role in load-bearing behavior [22,33].

Further analysis of the size-dependent energy distributions reveals distinct structural configurations across different sheet sizes (Fig. 2a and c, and Figs S6 and S7 in the Supporting Information). Larger sheets (such as *L* = 80 nm) mainly undergo extensive self-folding, producing a greater total area fraction of flat regions, $\sum {f}_{{\mathrm{area}}}$ (Fig. 2c(i)), and these flat regions are predominantly associated with low energy levels (Fig. 2b(i)). In contrast, smaller sheets (such as *L* = 30 nm) favor ridge-dominated structures with limited self-folding, leading to a reduced total area fraction of flat regions and an increased ${f}_{{\mathrm{area}}}$ in the high-energy regime. This structural differentiation underlies the negative size effect: ridge networks in smaller sheets store and distribute energy more effectively, providing superior mechanical resistance compared with the self-folded configurations of larger sheets. Such energy-distribution patterns are a general feature of confined 2D macromolecules, offering a mechanistic basis for the size-dependent mechanical behavior observed across material systems.

### Topological microstructure analysis

**Figure 3.**
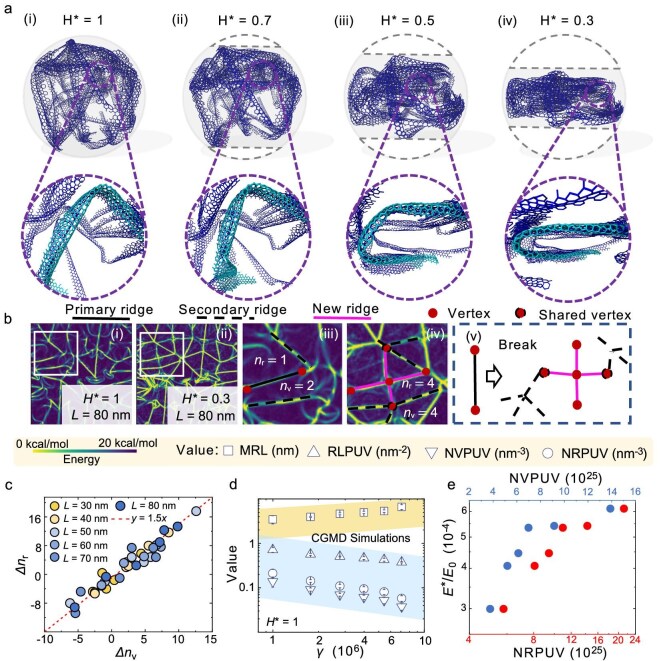
Topological evolution of ridge networks during densification of crumpled 2D macromolecules. (a) Sequential deformation of interwoven ridge structures, with an enlarged view of bending and folding process of one specific ridge structure (${H}^*$ = 1, 0.7, 0.5, 0.3). (b) Topological transformation analysis: (i and ii) Unfolded ridge network mappings of an $L = 80\ {\mathrm{nm}}$ film before (${H}^* = 1$) and after (${H}^* = 0.3$) uniaxial compression; (iii and iv) Enlarged highlighted regions (solid/dashed lines: primary/secondary ridges; filled dots: vertices; outlined filled dots: shared vertices); (v) Schematic of individual ridge evolution (${n}_{\rm r}$: ridge number; ${n}_{\rm v}$: equivalent vertex number). (c) Universal scaling between $\Delta {n}_{\rm r}$ (ridge increment) and $\Delta {n}_{\rm v}$ (vertex increment). (d) Statistical characterization of topological microstructures: MRL, RLPUV, NVPUV, NRPUV as a function of *γ*. (e) Correlation between load-bearing modulus and topological parameters.

To complement energy landscape analysis and explore tree limb-like ridge network growth, we performed in-depth topological microstructure analysis (adaptive potential energy-based threshold method, distinct from Fig. 2a’s fixed threshold) to clarify evolution under compression. This dynamic approach ensures optimal ridge visibility throughout compression, facilitating detailed structural tracking. As shown in Video S3, ridge structures form complex 3D interweaving networks during crumpling; representative analysis of a loading-direction-aligned ridge (light blue, Fig. 3a) reveals characteristic bending/folding deformations that contribute significantly to 3D crumpled particle load-bearing. This behavior aligns with prior findings where individual ridges initiate bending/folding at extremities upon reaching critical loading thresholds [21], with these localized deformations driving topological microstructure transitions during densification.

In multi-ridge crumpled particles, ridge network analysis reveals a systematic topological transformation mechanism governing structural evolution under deformation. As shown in Fig. 3b, an initial primary ridge (${n}_{\rm r} = 1$) and two equivalent vertices (${n}_{\rm v} = 2$) evolve into 4 ridges and 4 equivalent vertices via progressive deformation. Equivalent vertices include shared junctions (counted as 0.5 each) and full vertices, totaling 4. This topological transformation yields net incremental changes of $\Delta {n}_{\rm r} = 3$ and $\Delta {n}_{\rm v} = 2$ (Fig. 3b), establishing a universal ridge-to-vertex increment ratio of $\Delta {n}_{\rm r}/\Delta {n}_{\rm v} =$1.5. Physically, this reflects a local branching and reconstruction process in which existing ridges undergo instability, bending, and splitting during densification, generating more secondary ridges than newly formed vertices. CGMD simulations across sheet sizes and relative densities substantiate this relationship, with all data collapsing onto a master line with a slope of 1.5 (Fig. 3c). These results reveal that crumpled particles undergo continuous conformational transitions during densification, with additional ridge formation enhancing load-bearing capacity through a universal topological evolution pathway independent of sheet size or material properties. Densification catalyzes further ridge formation, establishing a dynamic feedback loop governing topological microstructure evolution.

To clarify the size-independent evolution of ridge networks in crumpled particles, we established a quantitative microstructural characterization framework. Four global metrics were defined to systematically access the microstructural features across initial sheet sizes: (1) Mean ridge length (MRL): the ratio of total ridge length to the number of ridges; (2) Ridge length per unit volume (RLPUV): the ratio of total ridge length to volume; (3) Number of ridges per unit volume (NRPUV): the ratio of the number of ridges to volume; and (4) Number of vertices per unit volume (NVPUV): the ratio of the number of vertices to volume. In CGMD simulations, ridge networks were extracted from the discrete honeycomb lattice structure through continuity analysis and image processing of the energy density map (refer to Materials and Methods). Statistical analysis of processed images enabled characterization of the four metrics.

Figure S4 shows NVPUV and NRPUV evolution with ${H}^*$ for crumpled particles of different sheet sizes, further confirming the universality of topological microstructure evolution across scales, wherein the relative size relationship of the topological load-bearing capacities is inherent and conserved at various scales. Figure 3d presents a statistical analysis of topological microstructures, where the MRL exhibits a positive correlation with sheet size (larger sheets generate progressively longer ridges during densification [43]). Conversely, RLPUV, NRPUV, and NVPUV demonstrate inverse relationships with sheet size, directly manifesting the characteristic negative size effect. These findings establish microstructural density variations as the fundamental determinant of load-bearing capacities in crumpled particles.

Figure 3e presents the relationship between mechanical properties of crumpled 2D macromolecules and topological microstructures. Load-bearing modulus increases with microstructural density, confirming mechanical performance modulation through microstructural density control. This directly explains the negative size effect: smaller sheets naturally form higher ridge densities during crumpling, enhancing load-bearing capacity. Using symbolic regression (Note S2), we derived an approximate relationship between *P* and microstructural density: $P = 0.128{\mathrm{RLPUV}}/{H}^*$. While RLPUV serves as the representative measure of microstructure density metric, equivalent representations could also be established using metrics NRPUV or NVPUV. As shown in Fig. S9b, the formula exhibits strong predictive power, reflecting direct proportionality between load-bearing pressure and microstructural density. This finding provides a quantitative framework linking microscale topological features to the macroscopic mechanical response of crumpled particles, establishing the fundamental mechanism underlying size-dependent behavior in crumpled 2D macromolecules. It is important to emphasize that the use of symbolic regression here is intended to explore the underlying analytical structure of the relationship between variables without presupposing any specific functional form. The emergence of such a compact expression, driven purely by data, therefore reflects the genuine physical simplicity of the underlying mechanism: microstructural density alone governs the topological load-bearing capacity of crumpled 2D macromolecules.

### Experimental validations

CGMD simulations establish a universal negative size effect in crumpled 2D macromolecules, independent of crumpled density and material parameters. To further validate the universality of this relationship, we performed systematic compression tests on crumpled films of diverse macroscopic materials: paper, aluminum foil, polydimethylsiloxane (PDMS), and silicone rubber. This cross-scale validation confirms both the effect’s universality across material classes and scale invariance (length scales from the molecular (simulation) to macroscopic (experiment) regimes). Detailed material properties are given in Table 1 and Materials and Methods.

**Table 1.** Material characteristics for four different materials in experiments.

Materials	Thickness *t*_0_ (μm)	Material density ${\rho }_0$ (g/cm^3^)	Young’s Modulus ${E}_0$ (Pa)
Paper	104.1 ± 0.3	0.7	1.2 × 10^9^ ± 3.6 × 10^5^
Aluminum foil	20.5 ± 1.0	2.6	2.1 × 10^9^ ± 1.7 × 10^6^
PDMS	245.3 ± 2.1	1.0	9.6 × 10^5^ ± 1.5 × 10^4^
Silicone rubber	76.7 ± 0.3	4.2	4.7 × 10^6^ ± 3.4 × 10^5^

**Figure 4.**
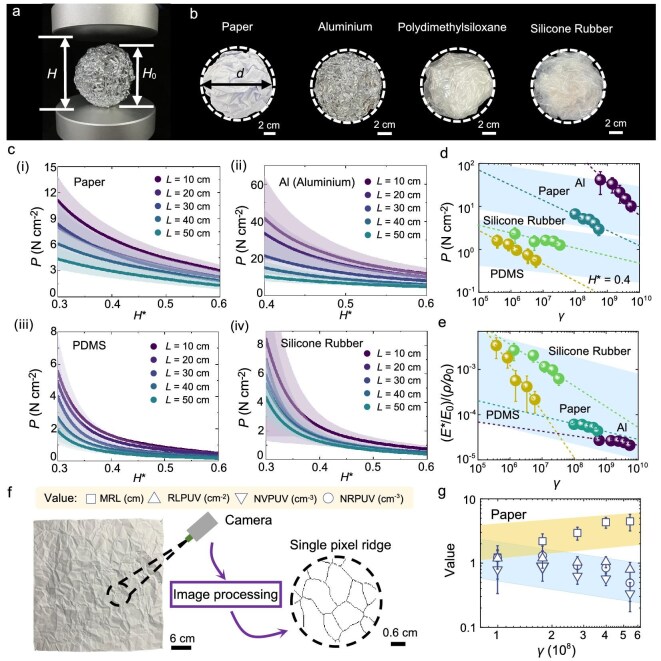
Experimental validation of the negative size effect across diverse materials. (a) Schematic of load-bearing capacity experiments. (b) Representative images of the four material systems in their uncompressed state. Here, ${H}_0$ equals to the diameter of the crumpled sphere. (c) Material-specific compressive *P–*${H}^*$ curves for (i) paper, (ii) aluminum foil, (iii) PDMS, and (iv) silicone rubber with varying initial film sizes. (d) Scaling relationship between *P* and $\gamma$ at ${H}^*$ = 0.4 across all material systems. (e) Relationship between dimensionless $( {{E}^*/{E}_0} )/( {\rho /{\rho }_0} )$ and$\ \gamma$, where ${E}_0$ and ${\rho }_0$ represent material-specific Young modulus and density, $\rho$ is the density of the crumpled ball. (f) Extraction methodology for ridge networks from unfolded crumpled paper samples through image processing techniques. (g) Statistical analysis of microstructural parameters in experimental samples as functions of $\gamma$ before uniaxial compression (${H}^*$ = 1).

Following established experimental protocols from previous studies [21,31,37,44], we replicated simulation conditions by manually compressing square films into spherical crumpled configurations (Fig. 4a and b). For each material, films of varying initial size *L* were compressed to target diameter $d = \sqrt {k{L}^3}$, where $k$ (material-specific constant unifying equivalent spherical diameter) was calibrated to 6.17 cm^−1^ (aluminum foil), 3.50 cm^−1^ (paper), 3.72 cm^−1^ (PDMS), and 4.16 cm^−1^ (silicone rubber). As shown in Fig. S10, each material demonstrated approximately equal crumpled densities $\rho$ across varying film sizes, though the relative densities ($\rho /{\rho }_0$) exhibited material-specific variations (Fig. S11 and Table S2). Additional experimental details are provided in Materials and Methods.

Analysis of *P–*${H}^*$ curves (Fig. 4c) (Each data point is the mean of 15 tests) reveal distinct size-dependent compression behaviors across the four material systems, yet consistently demonstrate an inverse correlation between *P* and film size. This universality validates our computational predictions of the negative size effect. Interestingly, plastic materials (aluminum foil and paper) exhibit gradual compression curves with progressive linearization at larger sizes, while elastic materials (PDMS and silicone rubber) display sharp pressure increases at ${H}^*$≈ 0.4. This divergent behavior stems from fundamental differences in their internal structural evolution during densification: elastic materials undergo systematic layer-by-layer stacking, establishing ordered arrangements with relatively larger interlayer spacing. Consequently, the compressive response remains smooth during the early densification stages (${H}^* = 0.4\sim 1$). Once these gaps are compacted (at ${H}^* \approx 0.4$), the load-bearing curves align with the behavior of layered materials, manifesting as a pronounced increase in curve slope [16,31,32,37,38,45]. Conversely, plastic materials develop less ordered crumpled structures with reduced gap compaction efficiency, resulting in continuous, gradual compression curves throughout the densification process (Fig. S12). Notably, the CGMD simulation parameters were specifically calibrated to emulate elastic material behavior, resulting in good agreement with the experiments, as evidenced by the similar trend in the $P \\!-\\! {H}^{\mathrm{*}}$ response observed for the PDMS and silicone rubber curves and the simulation results [21,31] (Fig. 1b).

We quantitatively evaluated size-dependent load-bearing characteristics across various materials through *γ*. As shown in Fig. 4d, at ${H}^*$ = 0.4, *P* consistently decreases with an increase in *γ* across all four materials. Figure 4e further demonstrates that ${E}^*$ follows an approximate power-law scaling relationship, decreasing as *γ* increases (see Fig. S13 for fitting details). These experimental observations substantiate the universal negative size effect predicted by CGMD simulations, establishing a fundamental scaling relationship that transcends material-specific properties in crumpled systems. It is worth noting that, while material constitutive behavior is important for the quantitative mechanical response, the negative size effect identified here is governed primarily by topology-controlled microstructural density rather than by any specific constitutive law.

To validate the microstructural characteristics predicted by simulations, we conducted a detailed ridge structure analysis using crumpled paper as a representative example [31,44,46]. Ridge networks were directly extracted through image processing of unfolded crumpled papers (Fig. 4f), with creases corresponding to ridges in our computational model. Here, crease refers to the residual visible trace left on the sheet after unfolding, whereas ridge denotes the localized structural feature in the crumpled configuration. It is worth noting that all lighting conditions and camera angles were kept constant to control variables, and the extraction of the creases was performed in a manner similar to the simulation (Fig. S15). While this analysis was primarily restricted to paper specimens due to the challenges in obtaining discernible crease networks from other materials—particularly aluminum foil, where minimal thickness precluded effective unfolding and analysis—the results provide compelling validation of our computational predictions.

Statistical analysis of ridge structures reveals consistent trends with CGMD simulations (Fig. 4g): the MRL exhibits a positive correlation with film size, while RLPUV, NRPUV, and NVPUV demonstrate inverse relationships with increasing film size. Despite inherent differences in temporal and spatial scales between experimental and computational approaches, both methodologies converge on a fundamental conclusion: the negative size effect in crumpled particles is primarily governed by microstructural density. This concordance substantiates the universal size-dependent mechanical response in crumpled systems, confirming the behavior transcends scales from molecular to macroscopic regimes and applies across diverse material compositions.

These experimental validations not only confirm our computational predictions but also establish the practical significance of our findings for engineering applications. The universality of the negative size effect across materials with vastly different chemical compositions, mechanical properties, and dimensional scales demonstrates a fundamental physical principle governing crumpled 2D structures—one that can be leveraged for designing materials with precisely tailored mechanical responses through microstructural control.

## DISCUSSION

This work establishes fundamental principles for 2D macromolecule mechanics, departing from traditional materials science by demonstrating that crumpled 2D macromolecules’ mechanical performance originates from topology—controllable via geometric design—rather than dominant chemical composition or crystalline structure. This elevates topology to a key determinant of material behavior in materials engineering.

To further examine the role of inter-sheet contact interactions, we performed additional CGMD simulations with varying inter-sheet Lennard-Jones interaction strength ${\varepsilon }_{\rm LJ}$ (Note S4). The results show that stronger adhesion partially suppresses the negative size effect but does not eliminate it within the parameter range considered here confirming the robustness of the observed ‘smaller is stronger’ behavior.

**Figure 5.**
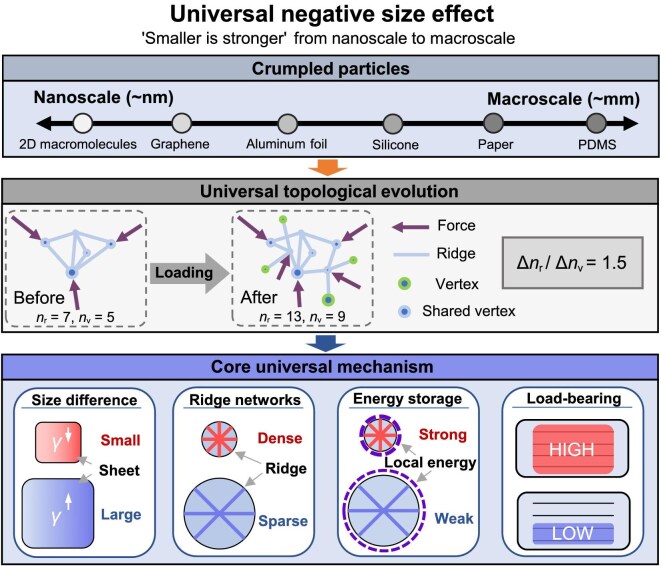
Universal mechanism of topological load-bearing in crumpled particles. Mechanical response is dominated by ridge structures. Smaller sheets naturally form higher ridge density, enhancing load-bearing capacity and yielding the negative size effect.

Integrating experiments with CGMD simulations, we comprehensively investigated topological load-bearing mechanisms of crumpled particles across scales, uncovering size-dependent mechanical responses in confined 2D systems. As summarized in Fig. 5, the negative size effect stems from three interconnected topological evolution mechanisms:

1. Ridges act as primary load-bearing scaffolds, with smaller sheets exhibiting higher ridge density to enable efficient energy distribution and structural reinforcement.
2. Microstructural density (spatial ridge distribution) governs the effect—smaller sheets form denser ridge networks per unit volume, while larger sheets undergo extensive self-folding with sparse ridges.
3. A universal topological evolution pathway (1.5 : 1 ridge-to-vertex increment ratio) conserves size-dependent mechanical advantages during densification, sustaining the ‘smaller is stronger’ relationship.

This study bridges microstructural topology and mechanical performance in confined 2D materials, providing core design principles for engineering crumpled particles via microstructural density control. The universality of these principles and scalability of crumpling support industrial applications in energy absorption and lightweight structures [47,48]. In addition, by quantifying topology-mechanics relationships, this research offers a framework for rational design of low-dimensional materials, independent of chemical composition. The universal negative size effect also provides insights into biological crumpling phenomena (e.g. insect wings, cell membranes) and suggests potential impacts of the 1.5 : 1 ridge-to-vertex ratio on electrical, thermal, and optical properties—meriting systematic investigation.

In conclusion, this study establishes a comprehensive framework linking microscale topological features to macroscopic mechanical responses in crumpled 2D macromolecules. By revealing the universal negative size effect and its underlying mechanisms, we provide fundamental design principles for engineering materials with precisely tailored load-bearing characteristics. The demonstration of scale-invariant behavior across diverse material systems, coupled with the identification of ridge density as the governing parameter, opens new avenues for rational design of advanced structural metamaterials. These findings not only deepen our understanding of confined 2D material mechanics but also establish a robust foundation for developing next-generation lightweight, high-performance structural components across applications ranging from energy absorption systems to biomedical devices.

## MATERIALS AND METHODS

### CGMD simulations

The CG model was developed based on graphene’s atomic structure, employing a 4:1 mapping scheme where each CG bead (mass: 48 g/mol) represents four carbon atoms [40,49] (Fig. S1). The force field of the CG model encompasses bond (${V}_{\rm b}$), angle (${V}_{\rm a}$), dihedral (${V}_{\rm d}$), and non-bonded interactions (${V}_{\rm nb}$), with parameters detailed in Table S1.

CGMD simulations were performed using the Large-scale Atomic/Molecular Massively Parallel Simulator (LAMMPS) under periodic boundary conditions in a 500 nm × 500 nm × 500 nm cubic simulation box. The system was initially energy-minimized using the conjugate gradient algorithm, followed by a 2 ns equilibration in the NVT ensemble at 300 K until potential energy convergence. Temperature regulation was achieved using the Nose–Hoover thermostat. The compression protocol consisted of two sequential stages: isotropic compression via virtual spherical shells (Fig. S1) and subsequent uniaxial compression between two virtual plates (Fig. S2) at a compression rate of 50 m/s. In each simulation (and experiment), a single sheet is crumpled into one spherical particle. Here, the spherical shape was chosen to remain consistent with previous studies and to facilitate control of variables such as relative density [31,32]. In addition, we carried out CGMD simulations on the prepared spherical particles along different loading directions and confirmed that anisotropy has a negligible effect on the mechanical response. The corresponding results are provided in Note S1 of the Supporting Information. During the simulations, the virtual spherical confinement was retained. This choice was made partly for consistency with previous studies [21,40], and partly to preserve the structural integrity of the crumpled configuration during the simulation so that the expected ridge network could be maintained. Details of this protocol choice are also discussed in Note S1. The integration time step was set at 3 fs throughout the simulations. To account for the stochastic nature of conformational densification transitions, all reported results represent averages from six independent simulations. In addition, we performed additional CGMD simulations to elucidate the effect of loading rate; details are provided in Note S1 of the Supporting Information.

### Experimental materials

Four representative thin film materials were investigated: paper, aluminum foil, PDMS, and silicone rubber. Commercial printing paper (80 g/m², thickness: 104.1 μm) exhibited Young’s modulus of 1.2×10^9^ Pa and bending stiffness of 1.1 × 10^−4^ J/m, maintained under controlled environmental conditions (35% relative humidity, room temperature). Aluminum foil (Reynouibs 615CF, thickness: 20.5 μm) possessed a modulus of 2.1 × 10^9^ Pa and bending stiffness of 1.5 × 10^−6^ J/m. The silicone rubber films (Gaodeyuan AA-01, Gaodeyuan Inc, China, thickness: 76.7 μm) demonstrated a modulus of 4.7 × 10^6^ Pa and bending stiffness of 1.8 × 10^−7^ J/m. PDMS films were fabricated using Sylgard 184 (Dow Corning, DC184) at a 10 : 1 base-to-curing agent ratio. The mixture was vacuum-degassed to eliminate air bubbles, cast onto polyacrylic acid-coated glass substrates, and thermally cured at 80°C for 2 hours under vacuum. The cured films were precisely excised, immersed in ultrapure water for detachment, and air-dried. Film thickness (245.3 μm), determined via confocal microscopy (Zeiss, LSM 880), yielded films with a modulus of 9.6×10^5^ Pa and bending stiffness of 1.2 × 10^−6^ J/m.

### Crumpling procedure

Square films were subjected to a standardized manual crumpling protocol to achieve uniform and stochastic conformational densification. The process is initiated with random corner-to-center folding, followed by multi-directional repeated compressions to form quasi-spherical configurations of desired density. The crumpled specimens were encapsulated in polymeric films to stabilize their morphology and facilitate precise radius measurements. To ensure experimental reproducibility and minimize systematic bias, the densification process was performed by multiple operators under controlled conditions using powder-free gloves. The final spherical radius (*R*) was determined by analyzing images taken from three orthogonal perspectives. The crumpled density was calculated as $\rho = 3{V}_{{\mathrm{film}}}/4\pi {R}^3$, where *R* is the average of the measured radii and ${V}_{{\mathrm{film}}}$ represents the volume of the original film. Statistical reliability was ensured through 15 independent trials for each material system.

### Compression test

The mechanical response of crumpled specimens was characterized using a universal testing machine (WANCE-ETM104B, Shenzhen Wance Equipment Co., Ltd, China) equipped with a force sensor capable of measuring forces in the range of 0–10 kN. Uniaxial compression tests were performed at a constant displacement rate of 5 mm/min until reaching a terminal load of 1 kN. Specimens were precisely centered between parallel indenters to ensure uniform load distribution. Statistical analysis was conducted on data averaged from 15 independent trials for each size.

### Extractions of ridge network

Topological characterization of ridge networks was performed through post-processing of experimental and simulation data sets, respectively. For CGMD simulations, atomic potential energy distributions were mapped onto the unfolded configuration. Cumulative elastic energy calculations yielded a grayscale image of the ridges, which was subsequently converted into a single-pixel image for statistical analysis (see Figs S14 and S15 for the detailed ridge extraction process). Experimental ridge analysis was conducted on flattened specimens using high-resolution digital imaging followed by multi-step image processing: creases detection via the Canny edge detection algorithm, contour recognition with Gaussian filtering [44,46], and skeletonization of the ridges through single-pixel processing [30].

The ridge network was decomposed through selective erosion at junction points, enabling individual ridge identification and measurement. Ridge lengths were quantified by pixel counting along each ridge, and then calibrated to the actual ridge lengths against known spatial dimensions. All distinguishable ridges were counted, with features below the characteristic ridge width excluded from the analysis.

## Supplementary Material

nwag305_Supplemental_Files

## References

[1] Liu Y, Yang M, Pang K et al. Environmentally stable macroscopic graphene films with specific electrical conductivity exceeding metals. Carbon 2020; 156: 205–11. 10.1016/j.carbon.2019.09.066

[2] Jaddi S, Malik MW, Wang B et al. Definitive engineering strength and fracture toughness of graphene through on-chip nanomechanics. Nat Commun 2024; 15: 5863. 10.1038/s41467-024-49426-3 38997272 PMC11245622

[3] Zhang DB, Akatyeva E, Dumitrică T. Bending ultrathin graphene at the margins of continuum mechanics. Phys Rev Lett 2011; 106: 255503. 10.1103/PhysRevLett.106.255503 21770654

[4] Liu H, Chen Y, Wang W et al. Homogenization of two-dimensional materials integrating monolayer bending and surface layer effects. J Mech Phys Solids 2025; 194: 105911. 10.1016/j.jmps.2024.105911

[5] Wang Y, Wang S, Li P et al. Conformational phase map of two-dimensional macromolecular graphene oxide in solution. Matter 2020; 3: 230–45. 10.1016/j.matt.2020.04.023

[6] Wang Y, Wang S, Gao Y et al. Determinative scrolling and folding of membranes through shrinking channels. Sci Adv 2024; 10: eadm7737. 10.1126/sciadv.adm7737 38669331 PMC11051672

[7] Yang Q, He X, Liu X et al. The effective properties and local aggregation effect of CNT/SMP composites. Compos B Eng 2012; 43: 33–8. 10.1016/j.compositesb.2011.04.027

[8] Silmore KS, Strano MS, Swan JW. Buckling, crumpling, and tumbling of semiflexible sheets in simple shear flow. Soft Matter 2021; 17: 4707–18. 10.1039/D0SM02184A 33978658

[9] Zhang CX, Brisson JA, Xu HJ. Molecular mechanisms of wing polymorphism in insects. Annu Rev Entomol 2019; 64: 297–314. 10.1146/annurev-ento-011118-112448 30312555

[10] McWhirter JL, Noguchi H, Gompper G. Deformation and clustering of red blood cells in microcapillary flows. Soft Matter 2011; 7: 10967–77. 10.1039/c1sm05794d

[11] Tomaiuolo G, Simeone M, Martinelli V et al. Red blood cell deformation in microconfined flow. Soft Matter 2009; 5: 3736–40. 10.1039/b904584h

[12] Dance A . The secret forces that squeeze and pull life into shape. Nature 2021; 589: 186–9. 10.1038/d41586-021-00018-x 33442045

[13] Llinares-Benadero C, Borrell V. Deconstructing cortical folding: genetic, cellular and mechanical determinants. Nat Rev Neurosci 2019; 20: 161–76. 10.1038/s41583-018-0112-2 30610227

[14] Lin YC, Torsi R, Younas R et al. Recent advances in 2D material theory, synthesis, properties, and applications. ACS Nano 2023; 17: 9694–747. 10.1021/acsnano.2c12759 37219929 PMC10324635

[15] Fomin VM . Topology-driven effects in advanced micro-and nanoarchitectures. In: Functional Nanostructures and Metamaterials for Superconducting Spintronics: from Superconducting Qubits to Self-Organized Nanostructures. Cham: Springer, 2018, 195–220.

[16] Fokker MC, Janbaz S, Zadpoor AA. Crumpling of thin sheets as a basis for creating mechanical metamaterials. RSC Adv 2019; 9: 5174–88. 10.1039/C8RA07565D 35514658 PMC9060670

[17] Li P, Wang S, Meng F et al. Conformational scaling relations of two-dimensional macromolecular graphene oxide in solution. Macromolecules 2020; 53: 10421–30. 10.1021/acs.macromol.0c01425

[18] Shohat D, Friedman Y, Lahini Y. Logarithmic aging via instability cascades in disordered systems. Nat Phys 2023; 19: 1890–5. 10.1038/s41567-023-02220-2

[19] Abbott AC, Buskohl PR, Joo JJ et al. Characterization of creases in polymers for adaptive origami structures. Smart Materials, Adaptive Structures and Intelligent Systems 2014; 46148: V001T01A009.

[20] Pradier C, Cavoret J, Dureisseix D et al. An experimental study and model determination of the mechanical stiffness of paper folds. J Mech Des 2016; 138: 041401. 10.1115/1.4032629

[21] Croll AB, Twohig T, Elder T. The compressive strength of crumpled matter. Nat Commun 2019; 10: 1502. 10.1038/s41467-019-09546-7 30944334 PMC6447532

[22] Lobkovsky A, Gentges S, Li H et al. Scaling properties of stretching ridges in a crumpled elastic sheet. Science 1995; 270: 1482–5. 10.1126/science.270.5241.1482

[23] Cerda E, Mahadevan L. Conical surfaces and crescent singularities in crumpled sheets. Phys Rev Lett 1998; 80: 2358–61. 10.1103/PhysRevLett.80.2358

[24] Gottesman O, Efrati E, Rubinstein SM. Furrows in the wake of propagating d-cones. Nat Commun 2015; 6: 7232. 10.1038/ncomms8232 26068220

[25] Giménez-Ribes G, Motaghian M, Linden Evd et al. Crumpled structures as robust disordered mechanical metamaterials. Mater Des 2023; 232: 112159. 10.1016/j.matdes.2023.112159

[26] Jayawardana WM, Liao Y, Li Z et al. Crumpled kirigami. Soft Matter 2023; 19: 1081–91. 10.1039/D2SM01584F 36722907

[27] Han F, Zhu Z. The mechanical behavior of foamed aluminum. J Mater Sci 1999; 34: 291–9. 10.1023/A:1004401521842

[28] Hanaor DA, Johnson EF, Wang S et al. Mechanical properties in crumple-formed paper derived materials subjected to compression. Heliyon 2017; 3: e00329. 10.1016/j.heliyon.2017.e00329 28653042 PMC5477149

[29] Martoïa F, Orgéas L, Dumont PJ et al. Crumpled paper sheets: low-cost biobased cellular materials for structural applications. Mater Des 2017; 136: 150–64. 10.1016/j.matdes.2017.09.031

[30] Vliegenthart GA, Gompper G. Forced crumpling of self-avoiding elastic sheets. Nat Mater 2006; 5: 216–21. 10.1038/nmat1581 16462740

[31] Croll AB, Liao Y, Li Z et al. Sticky crumpled matter. Matter 2022; 5: 1792–805. 10.1016/j.matt.2022.04.029

[32] Habibi M, Adda-Bedia M, Bonn D. Effect of the material properties on the crumpling of a thin sheet. Soft Matter 2017; 13: 4029–34. 10.1039/C6SM02817A 28512658

[33] DiDonna BA . Scaling of the buckling transition of ridges in thin sheets. Phys Rev E 2002; 66: 016601. 10.1103/PhysRevE.66.016601 12241494

[34] Lobkovsky AE . Boundary layer analysis of the ridge singularity in a thin plate. Phys Rev E 1996; 53: 3750–9. 10.1103/PhysRevE.53.3750 9964686

[35] Andrejevic J, Lee LM, Rubinstein SM et al. A model for the fragmentation kinetics of crumpled thin sheets. Nat Commun 2021; 12: 1470. 10.1038/s41467-021-21625-2 33674565 PMC7935925

[36] Kramer EM, Witten TA. Stress condensation in crushed elastic manifolds. Phys Rev Lett 1997; 78: 1303. 10.1103/PhysRevLett.78.1303

[37] Cambou AD, Menon N. Three-dimensional structure of a sheet crumpled into a ball. Proc Natl Acad Sci USA 2011; 108: 14741–5. 10.1073/pnas.1019192108 21873249 PMC3169141

[38] Lin YC, Sun JM, Yang HW et al. X-ray tomography of a crumpled plastoelastic thin sheet. Phys Rev E Stat Nonlin Soft Matter Phys 2009; 80: 066114. 10.1103/PhysRevE.80.066114 20365238

[39] Mirzaali MJ, Habibi M, Janbaz S et al. Crumpling-based soft metamaterials: the effects of sheet pore size and porosity. Sci Rep 2017; 7: 13028. 10.1038/s41598-017-12821-6 29026106 PMC5638806

[40] Liao Y, Li Z, Xia W. Size-dependent structural behaviors of crumpled graphene sheets. Carbon 2021; 174: 148–57. 10.1016/j.carbon.2020.12.006

[41] Lin J, Li P, Liu Y et al. The origin of the sheet size predicament in graphene macroscopic papers. ACS Nano 2021; 15: 4824–32. 10.1021/acsnano.0c09503 33682415

[42] Seung HS, Nelson DR. Defects in flexible membranes with crystalline order. Phys Rev A 1988; 38: 1005–18. 10.1103/PhysRevA.38.1005 9900464

[43] Bouaziz O, Masse JP, Allain S et al. Compression of crumpled aluminum thin foils and comparison with other cellular materials. Mater Sci Eng A 2013; 570: 1–7. 10.1016/j.msea.2013.01.031

[44] Blair DL, Kudrolli A. Geometry of crumpled paper. Phys Rev Lett 2005; 94: 166107. 10.1103/PhysRevLett.94.166107 15904253

[45] Cottrino S, Viviès P, Fabrègue D et al. Mechanical properties of crumpled aluminum foils. Acta Mater 2014; 81: 98–110. 10.1016/j.actamat.2014.07.069

[46] Gottesman O, Andrejevic J, Rycroft CH et al. A state variable for crumpled thin sheets. Commun Phys 2018; 1: 70. 10.1038/s42005-018-0072-x

[47] Meeussen AS, Van Hecke M. Multistable sheets with rewritable patterns for switchable shape-morphing. Nature 2023; 621: 516–20. 10.1038/s41586-023-06353-5 37730868

[48] Roh Y, Lee S, Won SM et al. Crumple-recoverable electronics based on plastic to elastic deformation transitions. Nat Electron 2024; 7: 66–76. 10.1038/s41928-023-01089-6

[49] Liao Y, Li Z, Chen L et al. Crumpling defective graphene sheets. Nano Lett 2023; 23: 3637–44. 10.1021/acs.nanolett.2c04771 36898061

