# Supplementary material for: Smaller is stronger: topological load-bearing of crumpled 2D macromolecule
Source: Natl Sci Rev. 2026 May 27;13(14):nwag305. doi: 10.1093/nsr/nwag305 (PMC13397538; doi:10.1093/nsr/nwag305)
Supplement: nwag305_Supplemental_Files [file nwag305_supplemental_files.zip › NSR_MS-2025-2470-Suppl.docx]

**Supplemental information**

Smaller is Stronger: Topological Load-bearing of Crumpled 2D Macromolecule

Runze Liang ^a=^, Kai Kang ^a=^, Huichao Liu ^a^, Yingbo Yan ^a^, Yan Chen ^a,^^[[1]](#footnote-1)^*, Yilun Liu ^a,*^

*Laboratory for multiscale mechanics and medical science, SV LAB, School of Aerospace, Xi'an Jiaotong University, Xi'an 710049, China*

Video S1. The video shows the crumpling of an elastic sheet with dimensions 30×30 nm.

Video S2. The video shows the uniaxial compression process of a crumpled matter with a radius of 4.7 nm.

Video S3. The video shows the deformation of a ridge structure in three-dimensional space under applied pressure.

Video S4. The video shows the deformation of a single ridge in three-dimensional space under applied pressure.

Video S5. The video shows the experimental preparation process of the crumpled paper ball.

Supplementary Materials Note S1

In order to overcome the limitations of the all-atomic model in both spatial and temporal aspects, we employed a coarse-grained (CG) model previously developed to simulate the wrinkling behavior of two-dimensional materials. Based on the atomic model of graphene, the CG model for graphene is derived using a 4-to-1 mapping scheme to preserve the geometric shape of the hexagonal lattice [1–4]. In this model, each CG bead with a mass of 48 g/mol represents four carbon atoms. The CG force field is established based on the principle of strain energy conservation, including bond interactions ($V_{b}$), angle interactions ($V_{a}$), dihedral interactions ($V_{d}$), and non-bonded interactions ($V_{nb}$). The functional forms and parameters of the CG force field are detailed in **Table S1**. This CG model not only effectively captures the mechanical behaviors of graphene, such as large deformations, interlayer anisotropic shear response, fracture, and nonlinear elastic deformation, but also significantly improves computational speed compared to all-atomic (AA) simulations, enabling larger spatiotemporal scales. The CG model has been applied successfully in molecular dynamics (MD) simulations, investigating structures such as multilayer graphene components, graphene-reinforced polymers, and nanostructured or architectured sheet assembly systems. More details on the development of the CG model can be found in previous studies.

| **Table S1**. Functional forms and interaction parameters of the sheet model. | | |
| --- | --- | --- |
| Interaction | Function form | Parameters |
| Bond | $V_{b}\left( d \right)=D_{0}{[1-e^{-\alpha(d-d_{0})}]}^{2}$  for $d<d_{cut}$ | $D_{0}=196.38kcal/mol$,  $300kcal/mol$  $\alpha=1.55Å^{-1}$  $d_{0}=2.8Å$  $d_{cut}=3.25Å$ |
| Angle | $V_{a}\left( \theta\right)=k_{\theta}{(\theta-\theta_{0})}^{2}$ | $k_{\theta}=409.4kcal/mol$  $\theta_{0}=120^{\circ}$ |
| Dihedral | $V_{d}\left( \phi\right)=k_{\phi}[1-cos(2\phi)]$ | $k_{\phi}=5kcal/mol, 20kcal/mol$ |
| Non-bonded | $V_{nb}\left( r \right)=4\varepsilon[\left( \frac{\sigma}{r} \right)^{12}-{(\frac{\sigma}{r})}^{6}]$  for $r<r_{cut}$ | $\varepsilon=0.001kcal/mol$  $\sigma=3.46Å$  $r_{cut}=12Å$ |

**
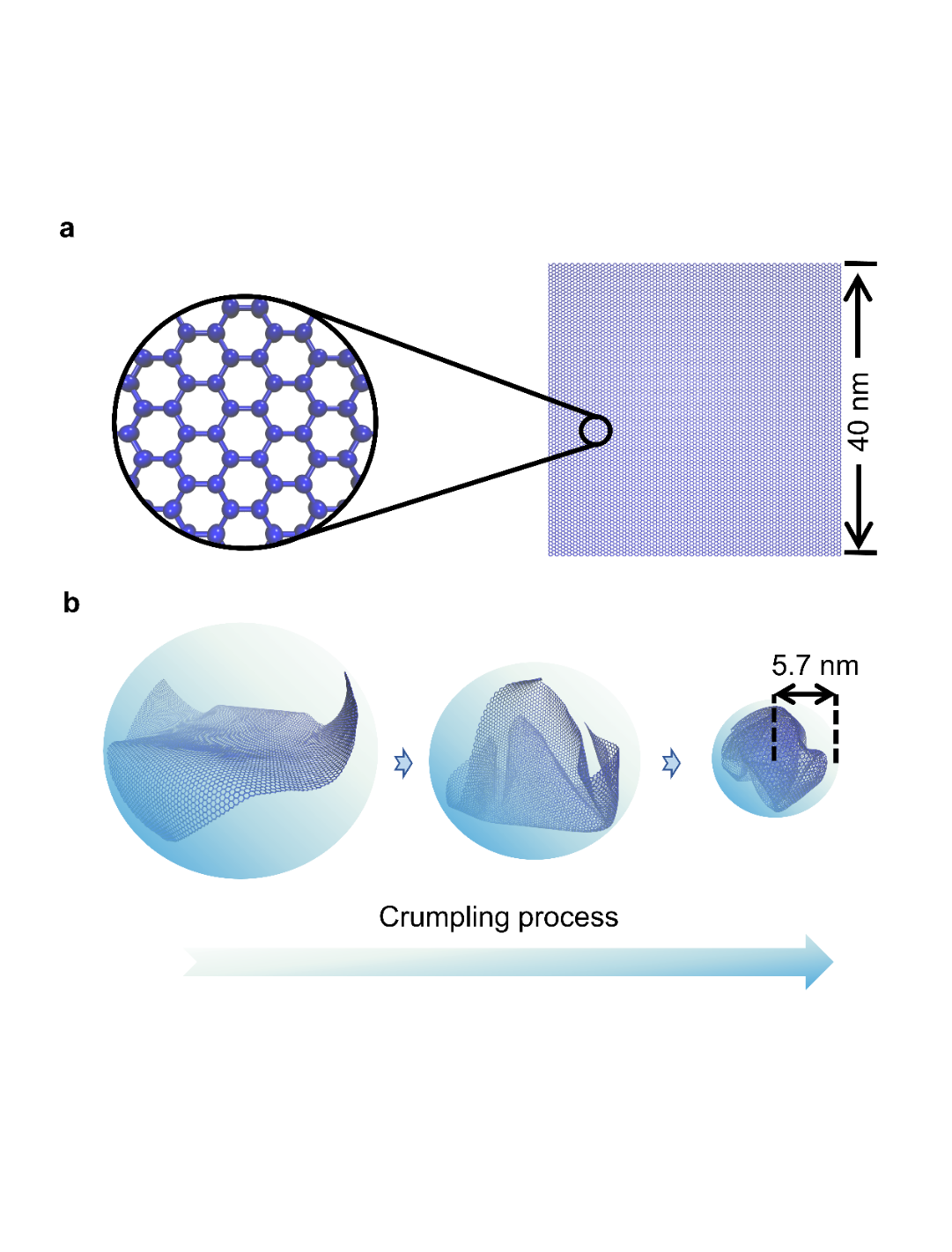
**

**Figure S1. Coarse-grained molecular dynamics model.** **(a)** 2D flat square film model with a hexagonal lattice composed of CG bead-spring elements with a 40 nm side length, 15936 beads in total, and a mass of 48 g/mol for each bead. **(b)** Schematic of the MD simulation of the crumpling process. Here, a confining sphere containing the film model is used to compress the sheet. As the radius of the confining sphere continuously decreases, the sheet gradually gets crumpled to the final sphere-like structure with a radius of 5.7 nm. The compression (uniaxial compression) simulation is performed based on this final crumpled sphere-like structure.

In MD simulations, before the compression simulation, a sequence of energy minimization, equilibration, and wrinkling simulations were performed. Initially, the thin film model was placed horizontally at the center of the simulation box with dimensions of 500 nm × 500 nm × 500 nm. Periodic boundary conditions were applied in all directions, and the simulation temperature was set to 300 K with a time step of 3 fs. Energy minimization was conducted using the iterative conjugate gradient algorithm, and a 2 ns NVT ensemble simulation was employed to equilibrate the system, ensuring that the total potential energy of the system converged to a nearly constant value. Subsequently, the wrinkling process was achieved by confining the thin film within a shrinking spherical volume. Specifically, when the sheet was inside the bounding sphere, a repulsive force ($F_{c}$) was applied to the sheet from the boundary of the sphere within a specified cutoff distance. The process of wrinkling the film involved reducing the radius ($R_{c}$) of the bounding sphere at a certain velocity (i.e., 50 m/s). The force applied at the edge of the sphere, $F_{c}$, can be expressed as:

$F_{c}\left( r_{i} \right)=\left\{ \begin{aligned} -K_{c}\left( r_{i}-R_{c} \right)^{2},for r_{i}\geq R_{c} \\ 0,for r_{i}<R_{c} \end{aligned} \right.$ (S1)

In the equation, $R_{c}$, $r_{i}$ and $K_{c}$ represent the bounding sphere radius, radial distance of the i-th sphere to the center of the bounding sphere, and the spring force constant, respectively. $K_{c}$ is set to 2.31 × 10^5^ kcal/mol/nm^3^, consistent with previous studies [5]. More details about the equilibrium and wrinkling simulations of the thin sheet can be found in earlier research [2,3]. All MD simulations were performed using the open-source software package Large-scale Atomic/Molecular Massively Parallel Simulator (LAMMPS) [6], and the visualization of MD simulations was accomplished through Visual Molecular Dynamics (VMD) [7].

**
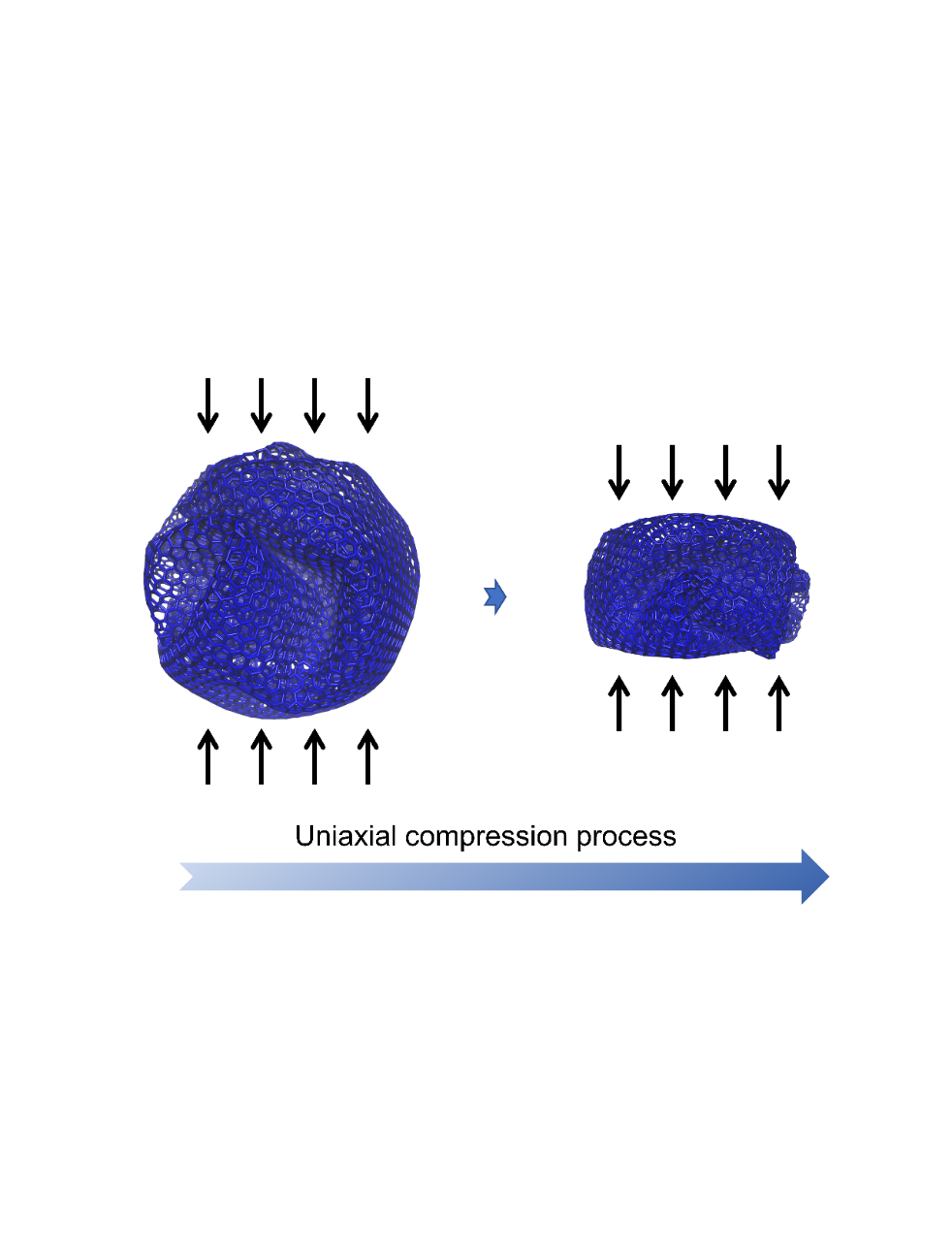
**

**Figure S2. Uniaxial compression of the CG model.**

After obtaining the crumpled film, we proceeded with uniaxial compression simulations. Initially, the crumpled film was placed between two virtual planes, with the distance between the virtual planes equal to the diameter of the crumpled film. Subsequently, compression simulations of the crumpled model were carried out by defining specific velocities for two opposing plates along distinct directions. In this study, we chose a compression velocity of 50 m/s, considering previous research indicating that the compression velocity has a negligible impact on the results [5].

**
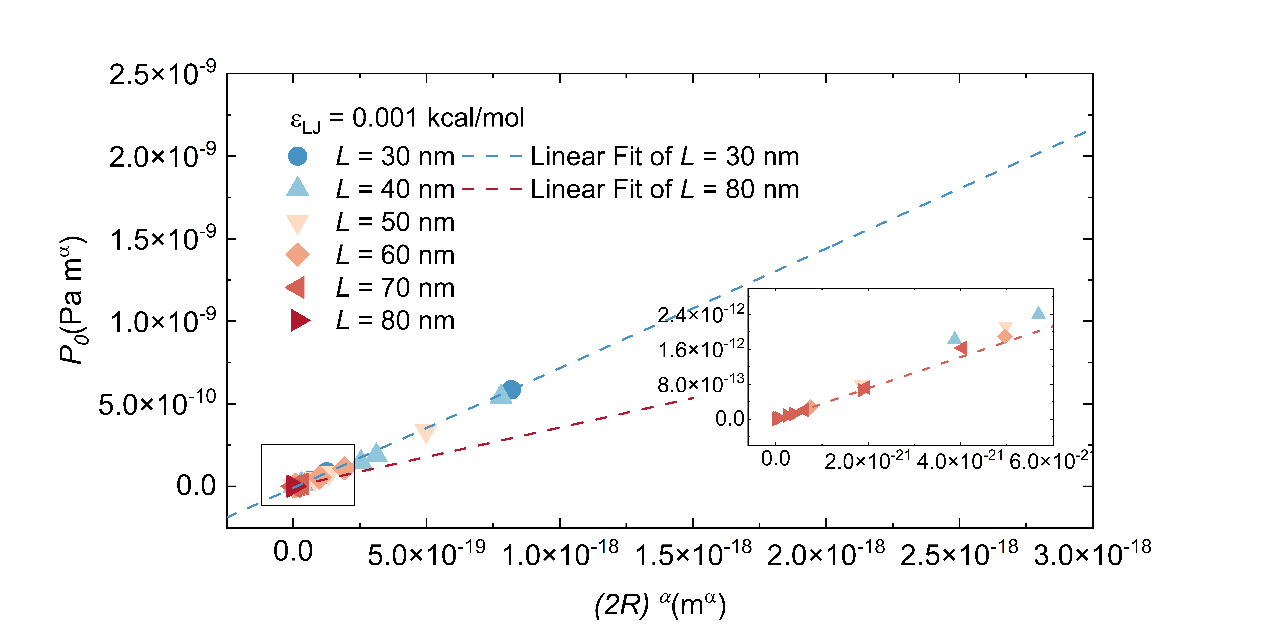
**

**Figure S3. Fitting of load-bearing modulus** $\boldsymbol{E}^{\boldsymbol{*}}$ **from simulations.** In the equation $P_{0}=E^{*}{(2R)}^{\alpha}$, $E^{*}$ is the slope of $P_{0}-{(2R)}^{\alpha}$ curves. The figure shows linear fitting curves for the maximum and minimum sizes (*D*_0_ = 196.38 kcal/mol, $k_{\theta}$ = 409.4 kcal/mol, $k_{\varphi}$ = 5 kcal/mol, and $\varepsilon$ = 0.001 kcal/mol). Note that, for quantitative values, we prefer to use the average of the $E^{*}$ value calculated directly for each data point.


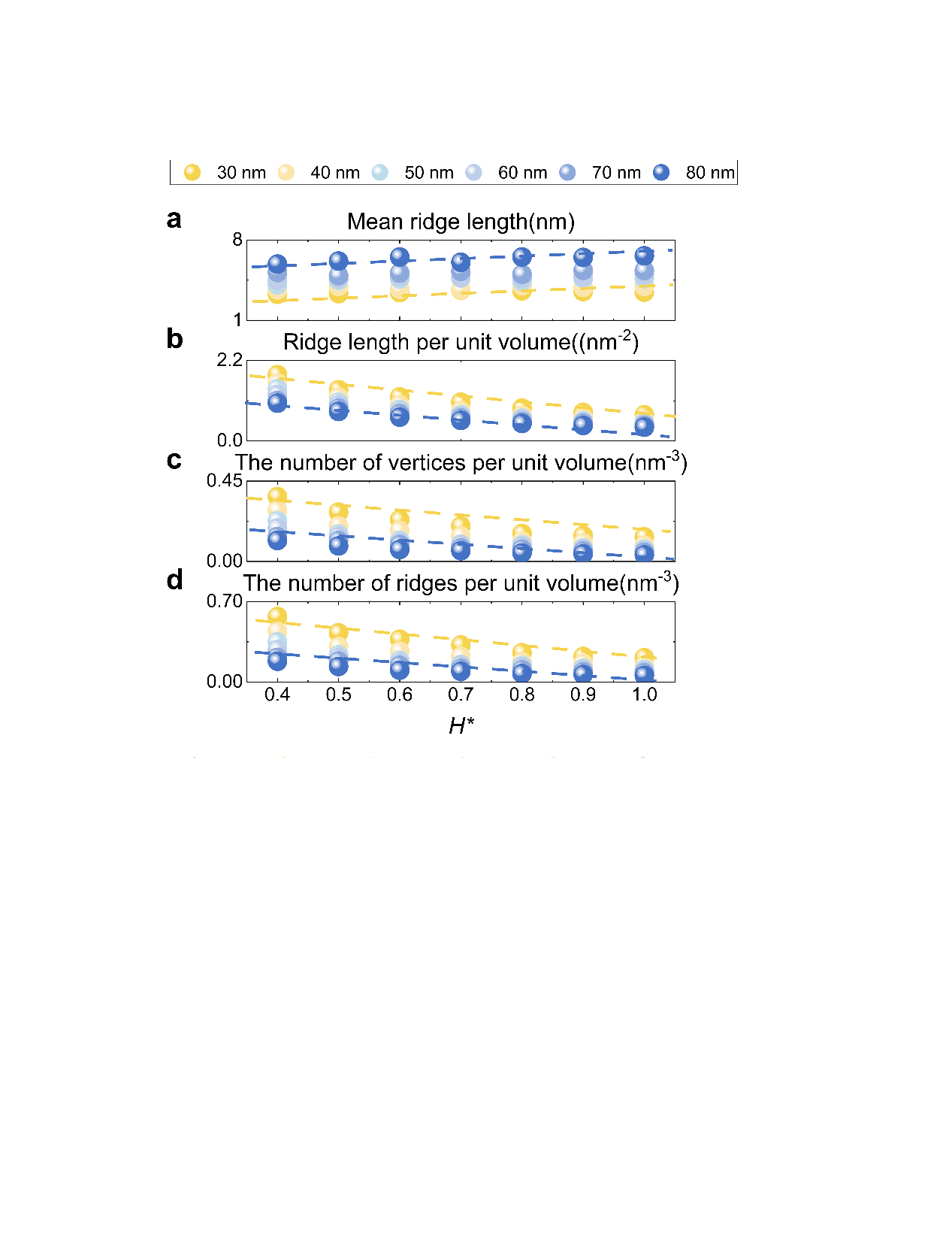


**Figure S4. The variations in four types of microstructure densities with the compression ratio for plates of different sizes. (a)** Mean ridge length. **(b)** Ridge length per unit volume. **(c)** Number of vertices per unit volume. **(d)** Number of ridges per unit volume.

Noting the discrepancy between the experimental and simulation loading rates v, and to rule out any possible influence of loading rate on our central finding of the negative-size effect, we performed additional MD simulations at *v* =5 m/s. As shown in **Figure S5**, both $P$ and $E^{*}$ still exhibit a negative size effect under low-rate loading, indicating that the loading rate does not affect our central finding.


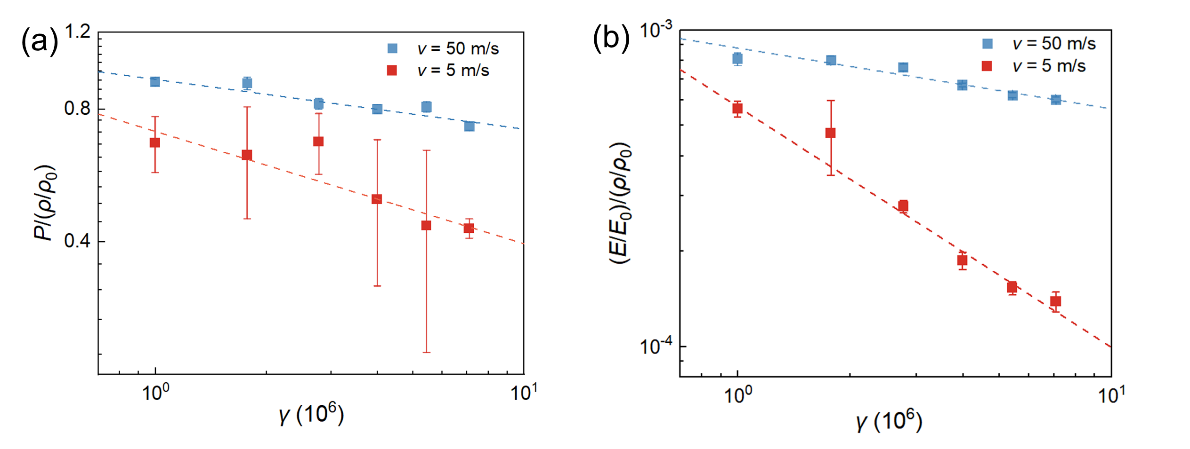


**Figure S5** **Scaling relationships at different loading rates** ($\rho/{\rho_{0}}=0.745$): (a) $P/(\rho/{\rho_{0})}$ versus $\gamma$ at $H^{*}$ = 0.4, and (b) $({E^{*}}/{E_{0}})/(\rho/{\rho_{0}})$ versus $\gamma$.

To justify the selection of 10 kcal/mol as the threshold, we performed sensitivity tests using thresholds of 8 kcal/mol and 12 kcal/mol. As shown in **Figure S6** and **S7**, the results remain consistent across these different values, indicating that our final conclusions are robust and independent of the specific threshold choice.


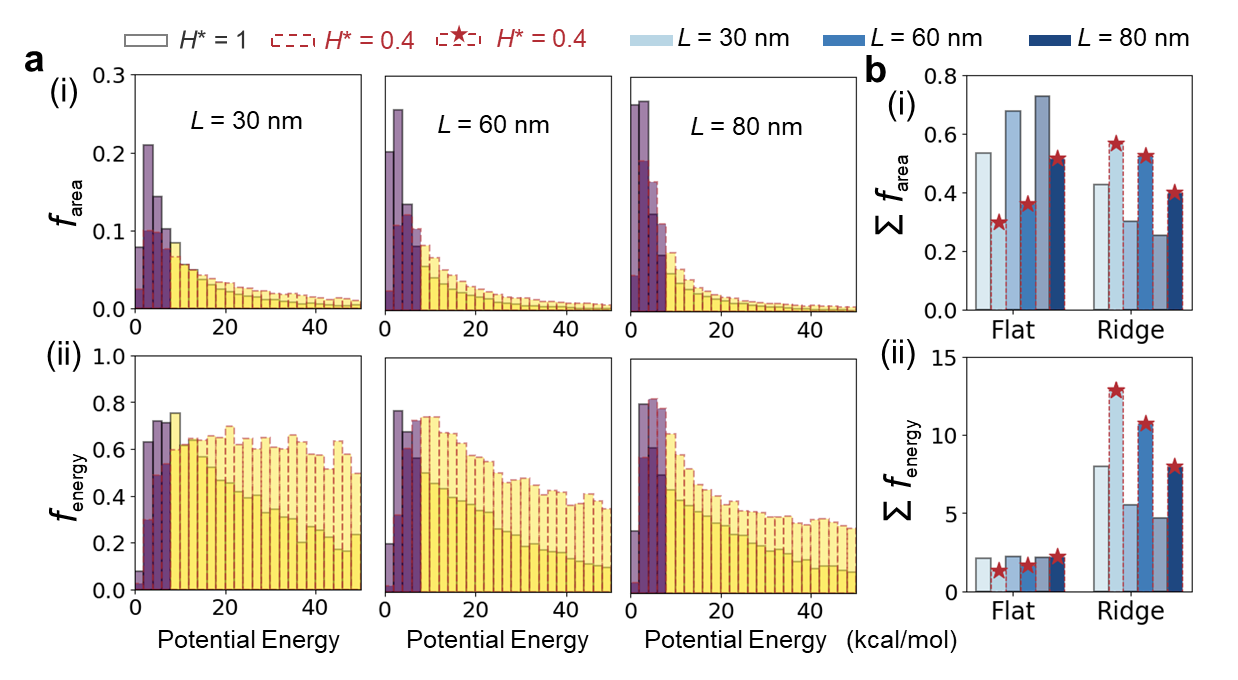


**Figure S6** **Quantitative characterization at a threshold of 8 kcal/mol**: (a) (i) the energy probability distribution *f*_area_ and (ii) the local energy density *f*_energy_ before compression ($H^{*}$ = 1) and after compression ($H^{*}$ = 0.4). (b) Comparative analysis of *f*_area_ and *f*_energy_ in flat and ridge structures across different sheet sizes.


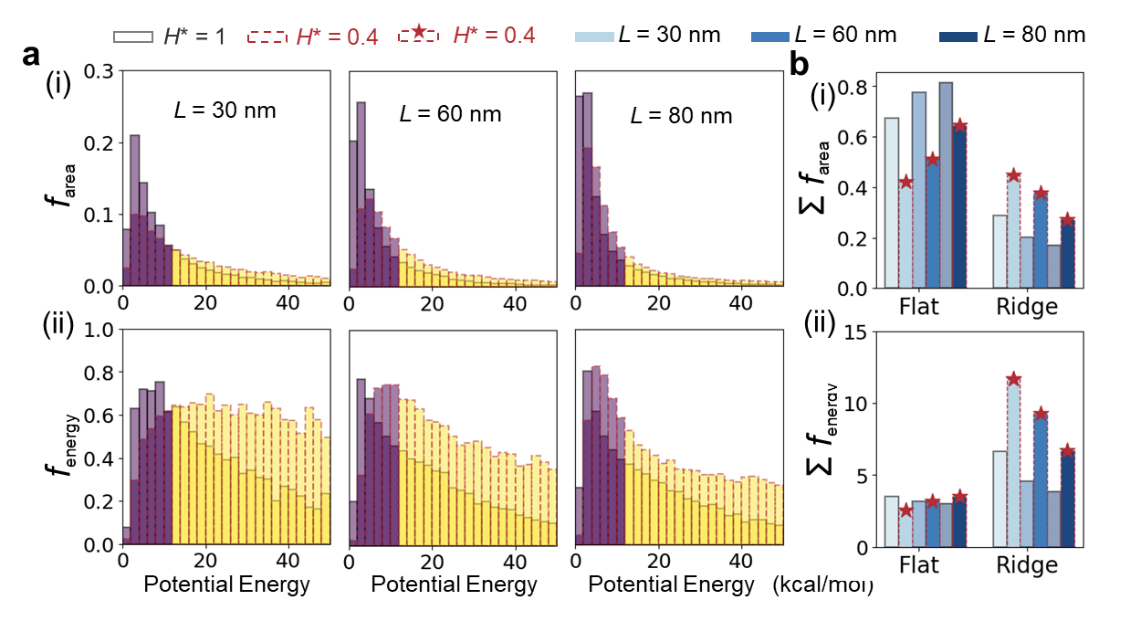


**Figure S7** **Quantitative characterization at a threshold of 12 kcal/mol**: (a) (i) the energy probability distribution *f*_area_ and (ii) the local energy density *f*_energy_ before compression ($H^{*}$ = 1) and after compression ($H^{*}$ = 0.4). (b) Comparative analysis of *f*_area_ and *f*_energy_ in flat and ridge structures across different sheet sizes.

Additional CGMD simulations were performed on three particles with different sizes. The resulting load-displacement curves are shown in **Figure S8**. For each particle size, the curves obtained along different directions exhibited slight anisotropy. It is worth noting, however, that the results reported in the main text are averaged values, which to some extent mitigate the influence of anisotropy.


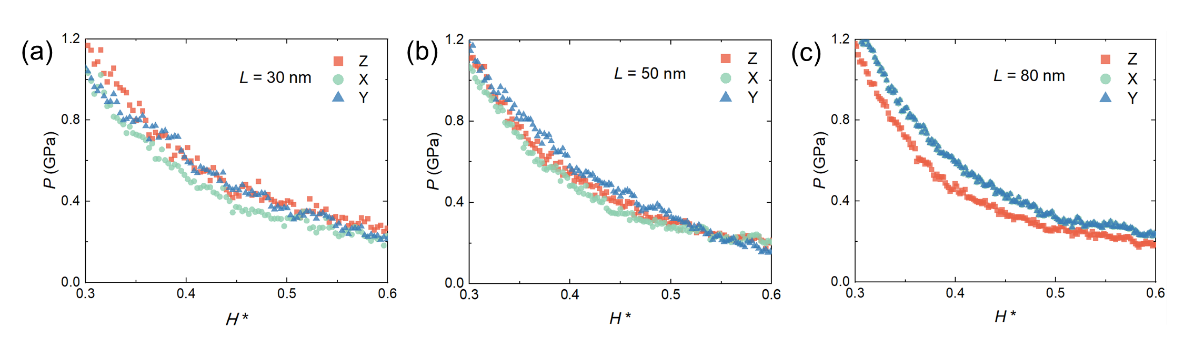


**Figure S8** ***P* - *H** curves of crumpled particles with different sizes** $\boldsymbol{L}$ **calculated by CGMD**: (a) $L = 30$ nm, (b) $L = 50$ nm, and (c) $L = 80$ nm.

Supplementary Materials Note S2

We used five variables that might influence the load-bearing capacity of crumpled materials (compression ratio, mean ridge length, ridge length per unit volume, number of vertices per unit volume, and number of ridges per unit volume) as parameters to train the symbolic regression model [8,9]. In this study, the symbolic regression model featured a population size of 5000 and evolved over 100 generations, with a tournament size of 20, aiming for a mean absolute error threshold of 0.001. To balance interpretability and complexity, we applied a parsimony coefficient of 0.01 and constrained constant values to the range (-1.0, 1.0). The initial tree depth ranged from 2 to 6, generated using a ‘grow’ method to create a diverse initial population. The functional set included basic arithmetic operations (‘add’, ‘sub’, ‘mul’) along with advanced functions (‘log’, ‘sqrt’, ‘div’) to capture non-linear relationships. The evolutionary process incorporated crossover (0.7 probability), subtree mutation (0.1), hoist mutation (0.1), and point mutation (0.1), with a point replace probability of 0.05, enabling thorough exploration of the solution space. We subsampled 90% of the data per generation to introduce variance and enhance model robustness. A fixed random state of 0 ensured reproducibility, and verbose output tracked the evolution across generations. The data for compression ratios $H^{*}=1, 0.8, 0.6, 0.4$ were selected as the training set, while the data for $H^{*}=0.7, 0.5$ were used to validate the accuracy of the symbolic regression model. As shown in **Figure S9a**, the result of the symbolic regression is $P=0.128\frac{x}{H^{*}}$, where *x* represents the ridge length per unit volume, and $H^{*}$ is the compression ratio. **Figure S9b** shows the model validation, indicating that the formula can effectively predict the load-bearing capacity of the structure.


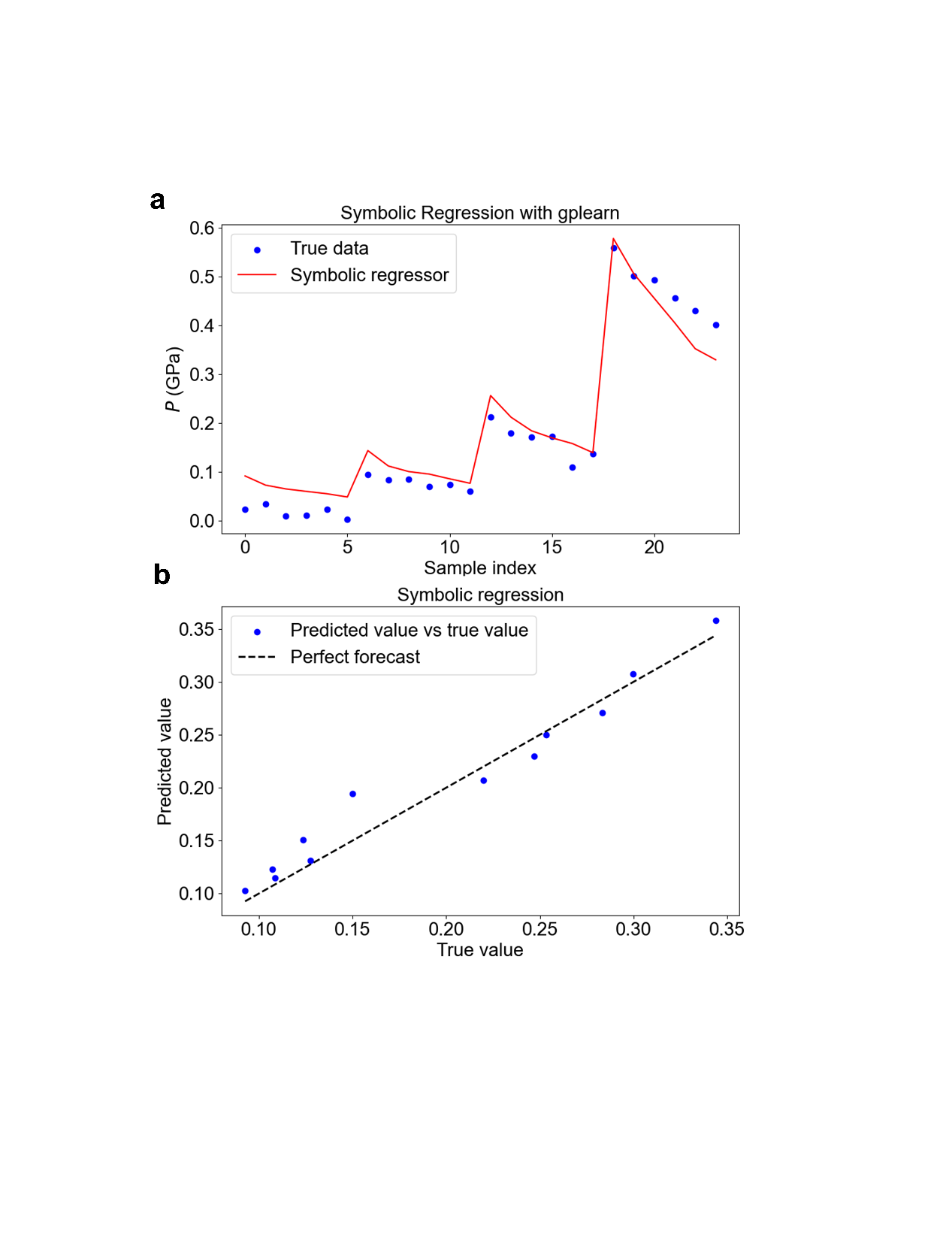


**Figure S9.** **Symbolic regression.** **(a)** The blue dots are the true values (*H** = 0.4, 0.6, 0.8, 1.0), and the red line is a formula fitted by symbolic regression using the true values, $P=0.128\mathrm{RLPUV}/{H^{*}}$, where $\mathrm{RLPUV}$ represents the ridge length per unit volume. **(b)** Comparison of predicted and true values ($H^{*}=0.5, 0.7$), and the black line indicates that the predicted value is equal to the true value.

Supplementary Materials Note S3

**
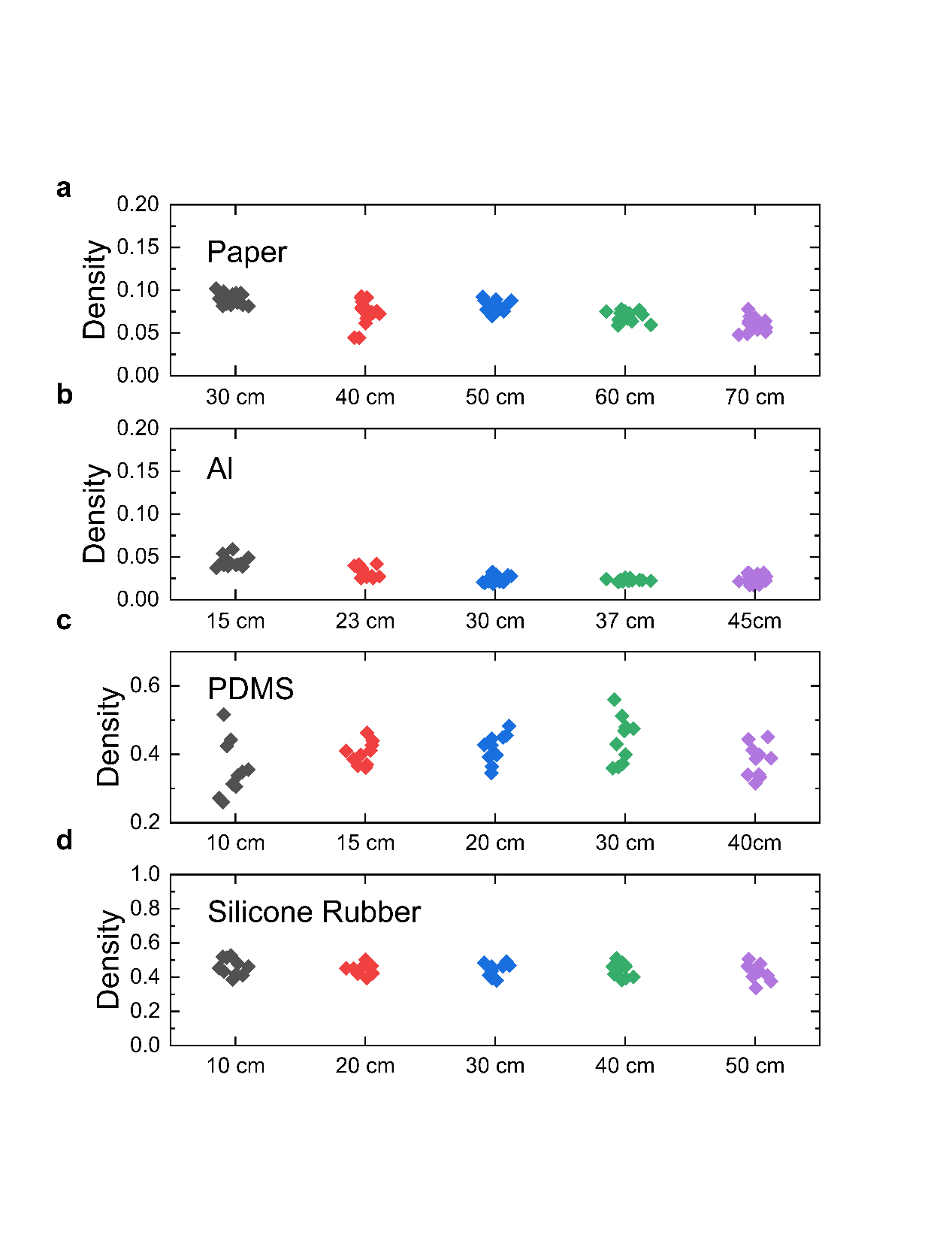
**

**Figure S10. The density distribution of the four materials used in the experiment before uniaxial compression.** **(a)** Paper. **(b)** Aluminum foil. **(c)** PDMS. **(d)** Silicone rubber. The unit of density is g/cm^3^

Paper, a common thin sheet in everyday life, exhibits relatively lower bending stiffness, resulting in the formation of rich irreversible crumpling. It is cost-effective and well-suited for studying the mechanical properties of crumpling. Similarly, aluminum foil, as a typical metallic thin sheet, shares properties similar to paper. Polydimethylsiloxane (PDMS), a commonly used experimental material in the field of soft solids, provides a material with easily verifiable material mechanics. Its modulus can be adjusted by varying the weight ratio of pre-polymer and cross-linking agent. PDMS has been employed in various related experiments. Silicone rubber, a commonly used industrial material, demonstrates excellent tensile properties, exhibiting noticeable differences in this experiment compared to the other three materials, allowing for a broader exploration of material characteristics. In the experiment, it is necessary to control the density of the same material to be the same. However, due to the manual crumpling process, it is challenging to precisely control the density to an identical value. Nevertheless, through multiple experiments, it has been ensured that the density distribution falls within a similar range, as shown in **Figure S10**. Therefore, it can be approximated that the impact of density can be considered negligible.

| **Table S2**. The average relative density of a material | | | | | |
| --- | --- | --- | --- | --- | --- |
| material | Paper | Al | PDMS | Silicone Rubber | |
| $\rho/\rho_{0}$ | \| 0.1386 \| \| --- \| | \| 0.3695 \| \| --- \| | \| 0.0255 \| \| --- \| | | \| 0.0226 \| \| --- \| |


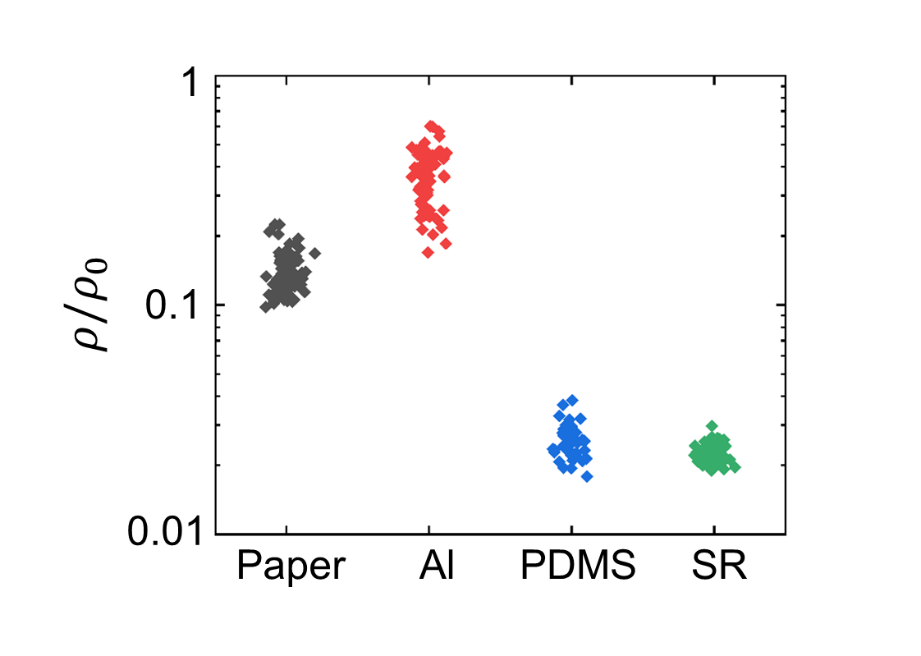


**Figure S11:** The relative density distribution of the four materials used in the experiment before uniaxial compression.


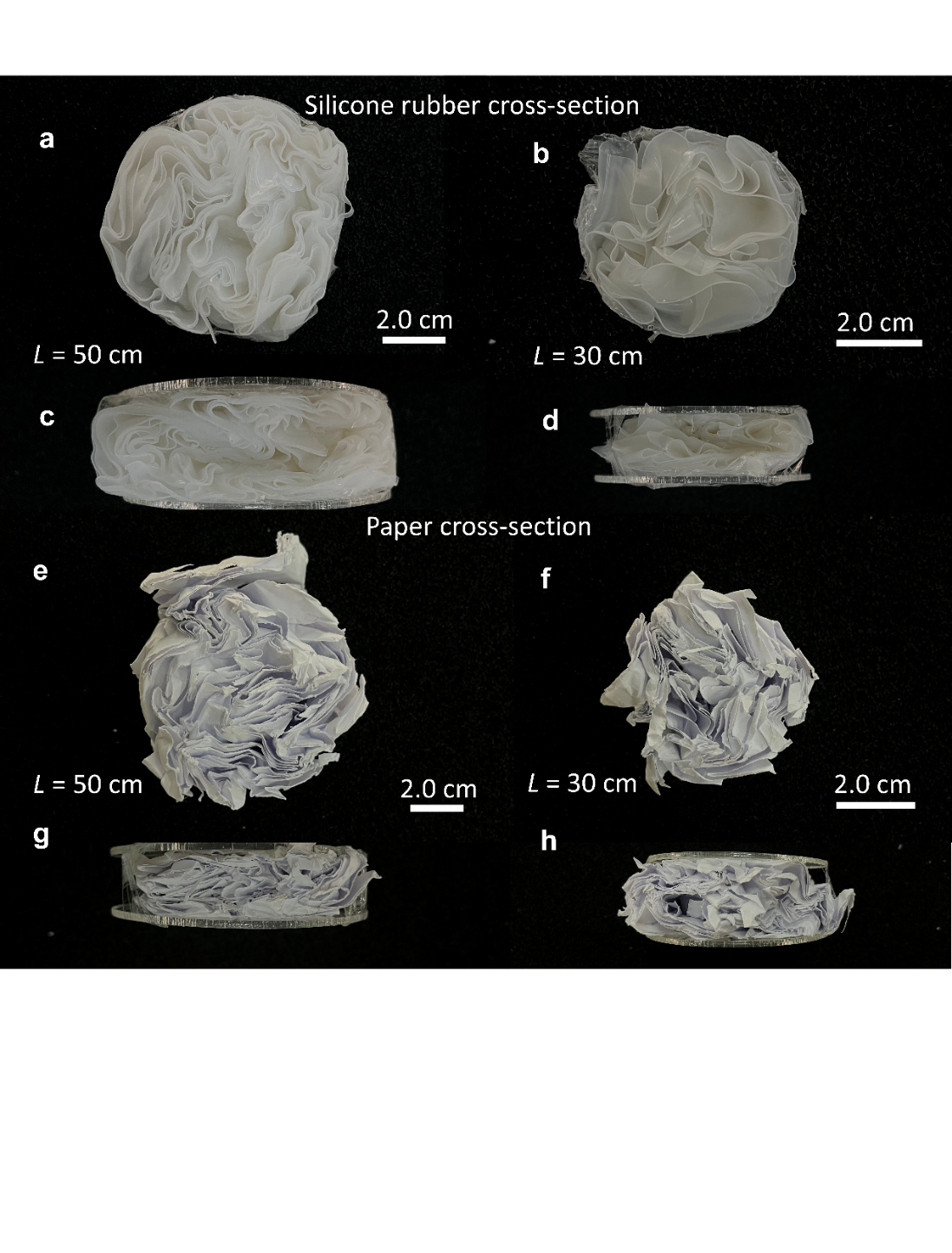


**Figure S12. Cross-section of the two materials in the experiment, silicone rubber and paper. (a)-(d)** Cross-sectional views of silicone rubber in two different sizes following compression (*L* = 50 cm, 30 cm). **(e)-(h)** Cross-sectional views of paper in two different sizes after compression (*L* = 50 cm, 30 cm).


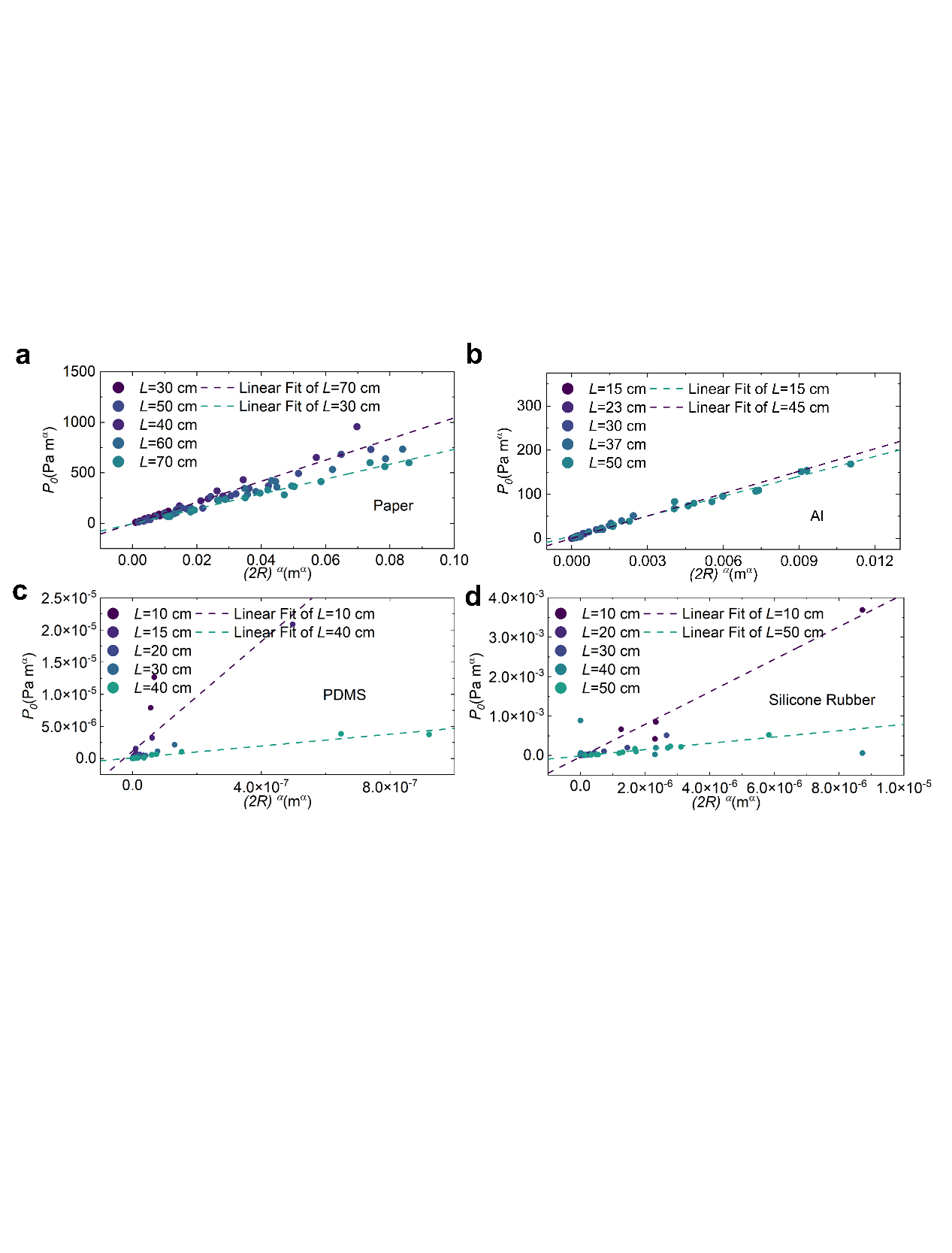


**Figure S13. Fitting of load-bearing modulus** $\boldsymbol{E}^{\boldsymbol{*}}$ **from the experiment.** In the equation $P_{0}=E^{*}{(2R)}^{\alpha}$, $E^{*}$ is the slope of $P_{0}-{(2R)}^{\alpha}$ curves. The figure shows linear fitting curves for the maximum and minimum sizes of four different materials. **(a)** Paper. **(b)** Aluminum foil. **(c)** PDMS. **(d)** Silicone rubber. Note that, for quantitative values, we prefer to use the average of the $E^{*}$ value calculated directly for each data point.

**
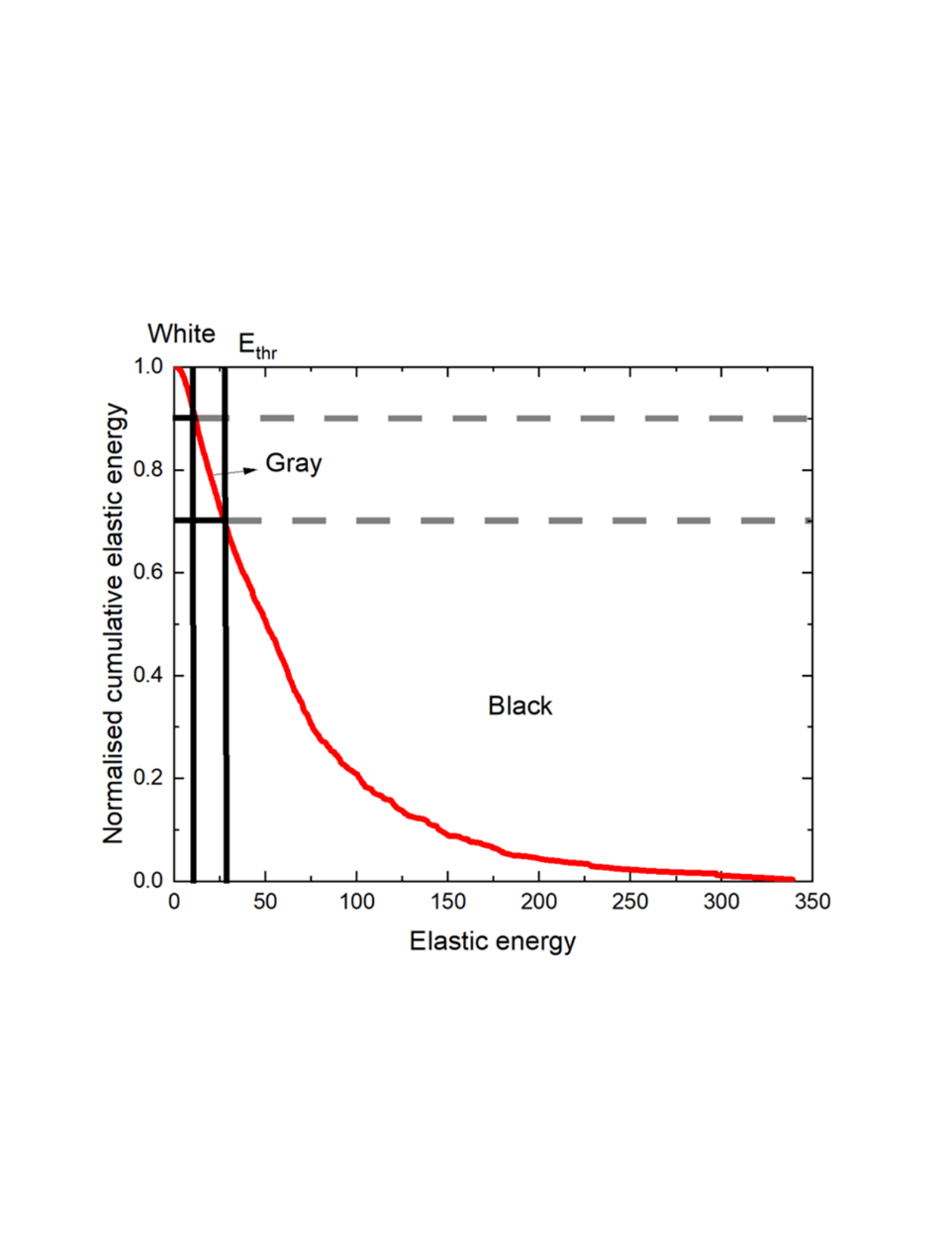
**

**Figure S14. Cumulative elastic energy.** Vertices are colored black if they contribute to the highest 10% of the total elastic energy, white if they contribute to the lowest 70% of the total elastic energy, and gray if they contribute to the middle 20% of the elastic energy.

**
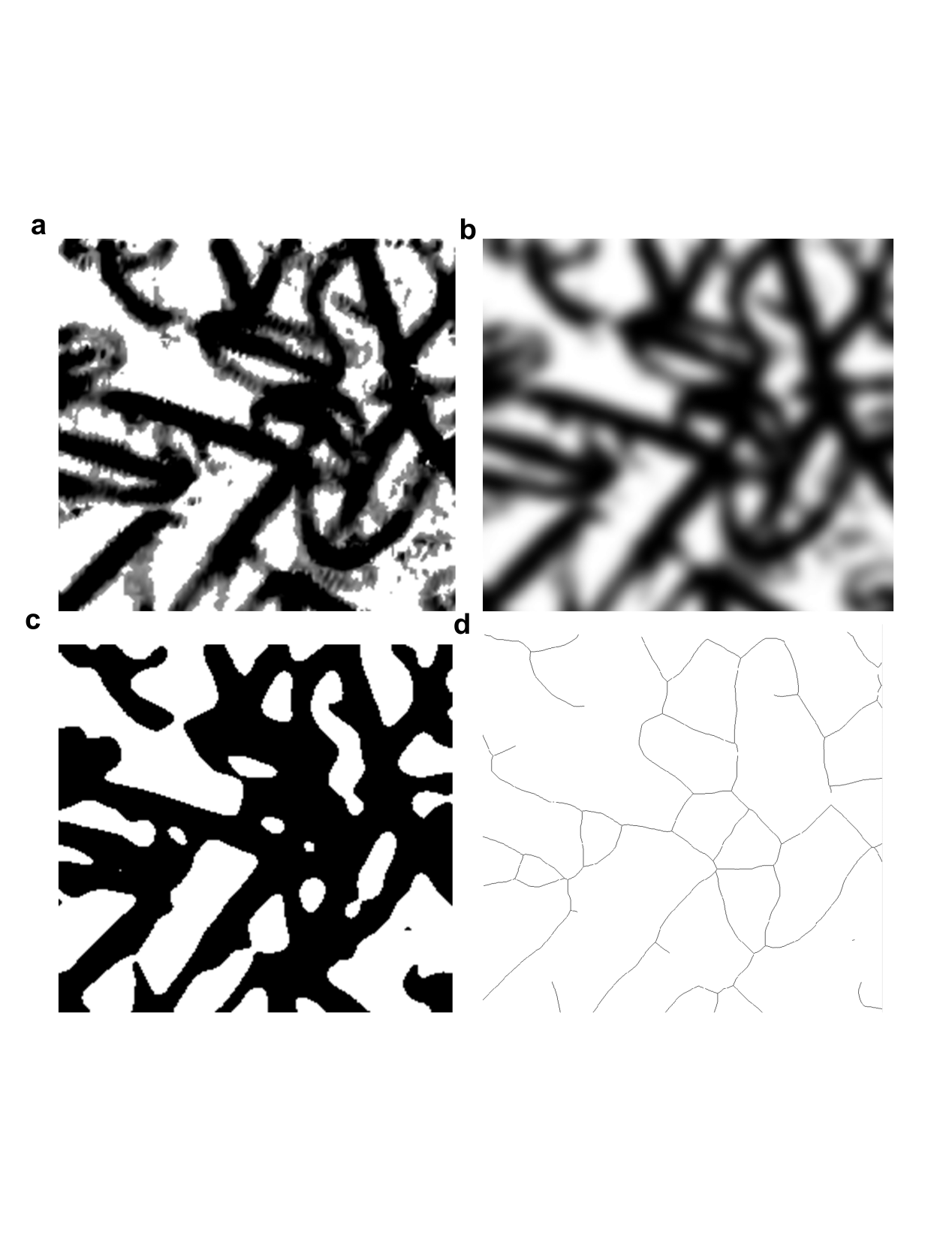
**

**Figure S15. Extractions of the ridge network by image processing**. **(a)** Energy density map of the raw image, **(b)** Image after Gaussian filter, **(c)** Thresholded image, **(d)** Skeletonized image in which junctions are eroded. It is worth noting that, during experimental image acquisition, all lighting conditions and camera angles were kept constant to ensure controlled variables, and crease extraction was performed in a manner consistent with the simulation.

Supplementary Materials Note S4

**Figure S16** shows the normalized load-bearing modulus $({E^{*}}/{E_{0}})/(\rho/{\rho_{0}})$as a function of sheet size *L* for different inter-sheet Lennard-Jones (LJ) interaction strengths $\varepsilon_{LJ}$. Increasing $\varepsilon_{LJ}$ leads to a higher overall modulus and a reduced slope of the size dependence, indicating that adhesion partially suppresses, but does not remove, the negative size effect.


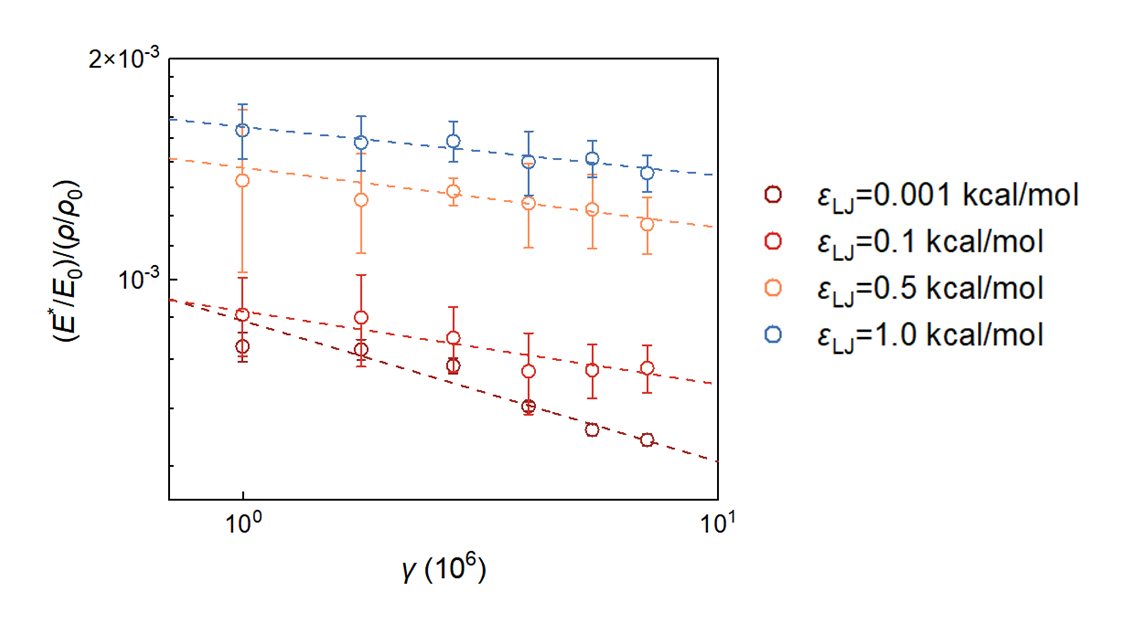


**Figure S16** **Effect of inter-sheet adhesion strength** $\boldsymbol{\varepsilon}_{\boldsymbol{LJ}}$on the relation between $({E^{*}}/{E_{0}})/(\rho/{\rho_{0}})$and γ following a scaling relationship obtained from CGMD simulations at $\rho/{\rho_{0}}=0.745$.

References

1. Ruiz L, Xia W, Meng Z *et al.* A coarse-grained model for the mechanical behavior of multi-layer graphene. *Carbon* 2015;**82**:103–15.

2. Liao Y, Li Z, Chen L *et al.* Crumpling defective graphene sheets. *Nano Letters* 2023;**23**:3637–44.

3. Liao Y, Li Z, Xia W. Size-dependent structural behaviors of crumpled graphene sheets. *Carbon* 2021;**174**:148–57.

4. Liao Y, Li Z, Ghazanfari S *et al.* Understanding the role of self-adhesion in crumpling behaviors of sheet macromolecules. *Langmuir* 2021;**37**:8627–37.

5. Croll AB, Liao Y, Li Z *et al.* Sticky crumpled matter. *Matter* 2022;**5**:1792–805.

6. Plimpton S. Fast parallel algorithms for short-range molecular dynamics. *Journal of computational physics* 1995;**117**:1–19.

7. Humphrey W, Dalke A, Schulten K. VMD: visual molecular dynamics. *Journal of molecular graphics* 1996;**14**:33–8.

8. Schmidt M, Lipson H. Distilling free-form natural laws from experimental data. *science* 2009;**324**:81–5.

9. Koza JR. Genetic programming as a means for programming computers by natural selection. *Statistics and computing* 1994;**4**:87–112.

1. = These authors contributed equally to this work.

   * Corresponding authors. [yanchen@xjtu.edu.cn](mailto:yanchen@xjtu.edu.cn) (Y. Chen), [yilunliu@mail.xjtu.edu.cn](mailto:yilunliu@mail.xjtu.edu.cn) (Y. Liu) [↑](#footnote-ref-1)
